# Bridging the gap between prostate radiology and pathology through machine learning

**DOI:** 10.1002/mp.15777

**Published:** 2022-06-13

**Authors:** Indrani Bhattacharya, David S. Lim, Han Lin Aung, Xingchen Liu, Arun Seetharaman, Christian A. Kunder, Wei Shao, Simon J. C. Soerensen, Richard E. Fan, Pejman Ghanouni, Katherine J. To'o, James D. Brooks, Geoffrey A. Sonn, Mirabela Rusu

**Affiliations:** ^1^ Department of Radiology Stanford University School of Medicine Stanford California USA; ^2^ Department of Urology Stanford University School of Medicine Stanford California USA; ^3^ Department of Computer Science Stanford University Stanford California USA; ^4^ Department of Biomedical Data Science Stanford University School of Medicine Stanford California USA; ^5^ Department of Electrical Engineering Stanford University Stanford California USA; ^6^ Department of Pathology Stanford University School of Medicine Stanford California USA; ^7^ Department of Epidemiology and Population Health Stanford University School of Medicine Stanford California USA; ^8^ Department of Radiology VA Palo Alto Health Care System Palo Alto California USA

**Keywords:** aggressive versus indolent cancer, cancer labels, deep learning, digital pathology, prostate MRI

## Abstract

**Background:**

Prostate cancer remains the second deadliest cancer for American men despite clinical advancements. Currently, magnetic resonance imaging (MRI) is considered the most sensitive non‐invasive imaging modality that enables visualization, detection, and localization of prostate cancer, and is increasingly used to guide targeted biopsies for prostate cancer diagnosis. However, its utility remains limited due to high rates of false positives and false negatives as well as low inter‐reader agreements.

**Purpose:**

Machine learning methods to detect and localize cancer on prostate MRI can help standardize radiologist interpretations. However, existing machine learning methods vary not only in model architecture, but also in the ground truth labeling strategies used for model training. We compare different labeling strategies and the effects they have on the performance of different machine learning models for prostate cancer detection on MRI.

**Methods:**

Four different deep learning models (SPCNet, U‐Net, branched U‐Net, and DeepLabv3+) were trained to detect prostate cancer on MRI using 75 patients with radical prostatectomy, and evaluated using 40 patients with radical prostatectomy and 275 patients with targeted biopsy. Each deep learning model was trained with four different label types: pathology‐confirmed radiologist labels, pathologist labels on whole‐mount histopathology images, and lesion‐level and pixel‐level digital pathologist labels (previously validated deep learning algorithm on histopathology images to predict pixel‐level Gleason patterns) on whole‐mount histopathology images. The pathologist and digital pathologist labels (collectively referred to as pathology labels) were mapped onto pre‐operative MRI using an automated MRI‐histopathology registration platform.

**Results:**

Radiologist labels missed cancers (ROC‐AUC: 0.75‐0.84), had lower lesion volumes (~68% of pathology lesions), and lower Dice overlaps (0.24‐0.28) when compared with pathology labels. Consequently, machine learning models trained with radiologist labels also showed inferior performance compared to models trained with pathology labels. Digital pathologist labels showed high concordance with pathologist labels of cancer (lesion ROC‐AUC: 0.97‐1, lesion Dice: 0.75‐0.93). Machine learning models trained with digital pathologist labels had the highest lesion detection rates in the radical prostatectomy cohort (aggressive lesion ROC‐AUC: 0.91‐0.94), and had generalizable and comparable performance to pathologist label‐trained‐models in the targeted biopsy cohort (aggressive lesion ROC‐AUC: 0.87‐0.88), irrespective of the deep learning architecture. Moreover, machine learning models trained with pixel‐level digital pathologist labels were able to selectively identify aggressive and indolent cancer components in mixed lesions on MRI, which is not possible with any human‐annotated label type.

**Conclusions:**

Machine learning models for prostate MRI interpretation that are trained with digital pathologist labels showed higher or comparable performance with pathologist label‐trained models in both radical prostatectomy and targeted biopsy cohort. Digital pathologist labels can reduce challenges associated with human annotations, including labor, time, inter‐ and intra‐reader variability, and can help bridge the gap between prostate radiology and pathology by enabling the training of reliable machine learning models to detect and localize prostate cancer on MRI.

## 1 INTRODUCTION

One in eight American men will be diagnosed in their lifetime with prostate cancer as per estimates from the American Cancer Society.^1^ In spite of clinical advances, prostate cancer remains the second deadliest cancer among men in the United States.^1^ Recent studies indicate that magnetic resonance imaging (MRI) greatly improves prostate cancer detection.^2^, ^3^ MRI–ultrasound fusion biopsies, used to target lesions outlined on MRI by radiologists, improve detection of clinically significant prostate cancer over ultrasound‐guided systematic biopsies alone^2^, ^4^, ^5^, ^6^, ^7^ As such, MRI is increasingly used to detect and localize prostate cancer, to guide targeted biopsies and in treatment planning.^8^

Despite the potential of MRI in detecting prostate cancer, subtle differences between benign and cancerous tissue on MRI lead to false negatives,^2^, ^4^ false positives^2^ and high inter‐reader variability^9^, ^10^, ^11^ among radiologists. Radiologist‐assigned Prostate Imaging‐Reporting and Data System (PI‐RADS) scores also suffer from wide variability, leading to missing or over‐calling aggressive cancers.^12^ Urologists and radiologists often recommend biopsy despite relatively low suspicion for cancer due to concerns for missed aggressive cancers. Moreover, MRI‐guided targeted biopsies are often supplemented with systematic biopsies, increasing morbidity (infection, bleeding, pain), as well as resulting in over‐treatment of indolent cancers. Accurate detection, localization, and aggressiveness characterization of all lesions on MRI can potentially assist clinicians in prostate cancer care by (1) guiding targeted biopsies to aggressive cancer, while reducing unnecessary biopsies for indolent cancers or benign regions of the prostate, and (2) deciding treatment planning based on location, extent, and aggressiveness of all lesions present (e.g., radical prostatectomy vs. focal therapy vs. active surveillance).

In order to standardize radiologist interpretations of prostate MRI, several machine learning‐based lesion detection methods have been developed to detect cancer, localize cancer, and characterize cancer aggressiveness using prostate MR images. As the goal of these automated lesion detection methods is to enable automatic evaluation of an entire MRI exam to provide a physician with outlines of all areas that are suspicious for cancer, these methods need to be trained with accurate labels with precise cancer location and extent. Prior machine learning methods for prostate cancer detection include traditional machine learning^13^, ^14^, ^15^, ^16^ as well as deep learning models using MRI.^17^, ^18^, ^19^, ^20^, ^21^, ^22^ The prior studies for automated prostate cancer detection and localization on MRI not only differ in the models used, but also in the ground truth labels used to train their models (Table 1).

**TABLE 1:** Summary of prior machine learning methods for prostate cancer detection and localization on MRI

Prior study	Method	Label type	Pathology confirmation	Pathology type	Mapping from pathology to MRI, if applicable
Saha et al.^22^	DL (U‐Net variant + residual classifier)	Radiologist	No	N/A	N/A
Yu et al.^23^	DL (ResNet + Panoptic FPN + Mask R‐CNN + Attention module)	Radiologist	No	N/A	N/A
Hosseinzadeh et al.^24^	DL (U‐Net variant)	Radiologist	No	N/A	N/A
McGarry et al.^16^	TML (Radiomics, Otsu thresholding)	Pathologist	Yes	RP	Semi‐automated MRI‐histopathology registration
De Vente et al.^41^	DL (U‐Net variant)	Semi‐automated region growing from targeted biopsy centroid	Yes	Targeted biopsy	Biopsy‐core coordinates
Sanyal et al.^19^	DL (U‐Net)	Radiologist	Yes	Targeted biopsy	Pathology reports
Sumathipala et al.^17^	DL (SPCNet variant)	Radiologist	Yes	RP and targeted biopsy	Cognitive registration or manually matching
Cao et al.^18^	DL (DeepLabV3+)	Radiologist	Yes	RP	Cognitive registration or manually matching
Bhattacharya et al.^20^	DL (SPCNet variant)	Pathologist	Yes	RP	Automated MRI‐histopathology registration
Seetharaman et al.^21^	DL (SPCNet)	Digital pathologist	Yes	RP	Automated MRI‐histopathology registration
Bhattacharya et al.^26^	DL (SPCNet variant)	Digital pathologist	Yes	RP	Automated MRI‐histopathology registration

Abbreviations: DL, deep learning; FPN, feature pyramid network; MRI, magnetic resonance imaging; PCa, prostate cancer; RP, radical prostatectomy; SPCNet, Stanford prostate cancer network; TML, traditional machine learning; N/A, not applicable.

The variety of labels used to train existing machine learning methods of prostate cancer detection using MRI include:

1. Radiologist outlines of PI‐RADS 3 or above lesions, without pathology confirmation^22^, ^23^, ^24^;
2. Radiologist outlines with pathology confirmation from targeted biopsy^19^;
3. Radiologist outlines with pathology confirmation from post‐operative whole‐mount histopathology images of radical prostatectomy patients through cognitive registration or manual matching^17^, ^18^;
4. Pathologist outlines on whole‐mount histopathology images mapped onto pre‐operative MRI through semi‐automatic or manual registration^16^;
5. Pathologist outlines on whole‐mount histopathology images mapped onto pre‐operative MRI using automated MRI‐histopathology registration^20^;
6. Gleason pattern labels on whole‐mount histopathology images derived from a previously validated deep learning algorithm^25^ mapped onto MRI through automated MRI‐histopathology registration^21^, ^26^;

Although different label types have been used in prior studies, no prior study investigated the comparative performance of the different label types to ascertain which labels provide the optimum training to machine learning methods applied to prostate MR images. All the label types used in prior studies have advantages as well as disadvantages. First, radiologist outlines without pathology confirmation are easier to obtain in large numbers from routine clinical care, but they include many false positives and may also miss cancers. Prior studies have shown that the false positive rate of radiologist outlines with PI‐RADS scores ≥3 can vary from 32% to 50%,^12^ depending on the experience of the radiologist. Moreover, radiologists can miss up to 12% of aggressive cancers during screening and 34% of aggressive cancers in men undergoing radical prostatectomy.^2^, ^4^ Second, radiologist outlines with pathology confirmation (through targeted biopsy) may still miss MRI‐invisible or hardly visible lesions and underestimate tumor extent.^27^ Third, cognitive registration or manual matching with post‐operative whole‐mount histopathology images of radical prostatectomy patients provides more accurate pixel‐level cancer mapping from histopathology images to pre‐operative MRI, but the cancer extent is still underestimated,^27^ and it is still challenging to outline the ~20% of tumors that are hardly visible or invisible on MRI.^10^ Fourth, pathologist labels mapped through registration onto MRI are the most accurate, but manual and semi‐automatic registration are labor intensive, time consuming, and require highly skilled experts in both radiology and pathology.^28^, ^29^, ^30^ Fifth, pathologist labels mapped onto MRI using automated MRI‐histopathology^31^, ^32^, ^33^, ^34^ registration can alleviate the challenges associated with manual or semi‐automatic registration approaches, but it is still challenging for human pathologists to annotate large datasets of whole‐mount histopathology images with pixel‐level annotations of cancer and Gleason patterns to train machine learning models on prostate MRI. Also, there can be variability in inter‐ and intra‐pathologist assignment of Gleason grade groups.

In this pilot study, we compare the different labeling strategies and analyze their effects in training machine learning methods for prostate cancer detection on MRI. Since a variety of machine learning model architectures have been used in existing studies, for simplicity of discussion, in this study, we use the general term “digital radiologists” to refer to all deep learning models that are applied to prostate MR images to detect and localize cancer. Similarly, for simplicity, we use the term “digital pathologists” to refer to all deep learning models applied to prostate histopathology images for detecting cancer and assigning Gleason patterns. We use the term “pathology labels” to collectively refer to labels on whole‐mount prostate histopathology images, derived either through human or digital pathologist annotations. To better understand the optimum approach for training reliable machine learning methods for prostate cancer, in this study, we seek answers to the following questions: (1) What effect does each label type have on the digital radiologist model they train? (2) What is the best way to train digital radiologist models? (3) Can digital pathologists be used to train reliable digital radiologists?

We hypothesize that digital pathologist annotations with pixel‐level histologic grade labels on whole‐mount histopathology images, when mapped onto MRI through automated MRI‐histopathology registration can (a) alleviate challenges associated with radiologist and pathologist labels, and (b) provide the most reliable digital radiologists for selective identification of aggressive and indolent prostate cancers. Recent studies have shown that digital pathologists have very high accuracy in Gleason grading on prostate histopathology images, and can significantly improve Gleason grading of pathologists by reducing variability in inter‐ and intra‐pathologist Gleason grade group assignment.^25^, ^35^, ^36^ Our prior SPCNet^21^ and CorrSigNIA^26^ studies are the only studies that used digital pathologist labels for training digital radiologists.

In order to study the effects of different labeling strategies on digital radiologists, we trained four different deep learning networks (SPCNet,^21^ U‐Net,^19^, ^37^ branched U‐Net,^26^ and DeepLabv3+^18^) commonly used for prostate cancer detection and localization in prior studies. For each network architecture, we trained four different digital radiologist models using 75 radical prostatectomy patients with four different types of labels: pathology‐confirmed radiologist labels ($L^{\mathrm{Rad}}$), pathologist labels mapped to MRI through automated registration ($L^{\mathrm{Path}}$), and two variants of digital pathologist labels mapped to MRI using automated registration, lesion‐level digital pathologist labels ($L_{\mathrm{Lesion}}^{\mathrm{DPath}}$) and pixel‐level digital pathologist labels ($L_{\mathrm{Pixel}}^{\mathrm{DPath}}$). Each label type selectively identified aggressive and indolent cancer on either a lesion level ($L^{\mathrm{Rad}}$, $L^{\mathrm{Path}}$, $L_{\mathrm{Lesion}}^{\mathrm{DPath}}$) or a pixel level ($L_{\mathrm{Pixel}}^{\mathrm{DPath}}$). Selective identification on a lesion level enables identifying entire lesions as aggressive or indolent, whereas selective identification on a pixel level enables identifying and localizing aggressive and indolent cancer components in mixed lesions. We evaluated our trained digital radiologists in two different patient cohorts (*N* = 315), including 40 men with radical prostatectomy and 275 men with targeted biopsies. Evaluation on two different cohorts enabled (1) comparing the effect of different labeling strategies on digital radiologist performance, and (2) testing the generalizability of the different models. Moreover, to ascertain if the effect of the labels is independent of the model type used, we used four different deep learning algorithms to train and evaluate our digital radiologists (SPCNet,^21^ U‐Net,^19^, ^37^ branched U‐Net, and DeepLabv3+^18^).

To summarize, the novel contributions of our study are:

1. We analyzed different labeling strategies to identify the best way to train digital radiologists for selective identification of aggressive and indolent prostate cancer using MRI.
2. We assessed the performance of digital pathologist labels and of the digital radiologists trained with these labels in comparison with human radiologist and pathologist labels.
3. We study whether the effect of different labeling strategies is independent of the model architecture.
4. We study whether the effect of different labeling strategies is consistent across different patient populations with different distributions of cancer.

## 2 MATERIALS AND METHODS

### 2.1 Data description

All data for this IRB‐approved retrospective chart review study was collected at Stanford University Medical Center. Two independent cohorts of subjects were used for this study. Cohort C1 comprised 115 patients who underwent radical prostatectomy, while cohort C2 included 275 men with or without prostate cancer who underwent MRI‐guided targeted biopsies for PI‐RADS scores ≥3 lesions.

Subjects in cohort C1 had a pre‐operative MRI prior to radical prostatectomy, and post‐operative whole‐mount histopathology images of the entire prostate. The median and inter‐quartile range for the number of days between the pre‐operative MRI and radical prostatectomy in cohort C1 were 90.25 and 68.8, respectively, while the mean and standard deviation were 103.3 and 82.2 days, respectively. The general slow growth of prostate cancer^38^, ^39^, ^40^ justifies using registration between MRI and histopathology images, even when the MRI is performed several months before surgery.

Subjects in cohort C2 had an MRI prior to biopsy which was used to guide the MRI‐transrectal TRUS fusion biopsy procedure.

#### 2.1.1 Magnetic resonance imaging

For subjects in both cohorts, multi‐parametric MRI scans were acquired using 3.0T GE MRI scanners with surface coils and without an endorectal coil. Axial T2‐weighted (T2w) MRI scans and apparent diffusion coefficient (ADC) maps derived from diffusion weighted images were used in this study (MRI acquisition characteristics are detailed in Table S1).

#### 2.1.2 Histopathology images

For patients in cohort C1, the prostates removed via radical prostatectomy were sectioned into slices using patient‐specific 3D‐printed molds generated from the pre‐operative MRI. The patient‐specific 3D‐printed molds enabled sectioning of the prostate into slices that were in the same plane as the T2w scans and had the same distance between slices. Each prostate histopathology image had a 4 μm thickness. After sectioning, the whole‐mount prostate slices were stained with hematoxylin and eosin (H&E), and scanned into a digital format with 20× magnification,^26^, ^31^ resulting in an in plane *X*–*Y* pixel size of 0.5 μm. For patients in cohort C2, biopsy samples were stained with H&E and subjected to pathological evaluation.

**Train–test splits**: The machine learning models were trained using 75 patients from cohort C1 in a five‐fold cross‐validation setting. The remaining 40 patients from cohort C1 and the entire cohort C2 (275 men) were used for independent testing of the models.

### 2.2 Labels

#### 2.2.1 Cancer and histologic grade labels

**Cohort C1**: Patients in cohort C1 had four different types of cancer labels. Each label type annotated each pixel of the prostate into one of the three classes: (1) normal tissue, (2) indolent cancer, and (3) aggressive cancer.

A previously validated deep learning model on histopathology images (henceforth called the “digital pathologist”)^25^ was used to predict Gleason patterns for each pixel of the prostate. Gleason pattern 3 predicted by the digital pathologist was considered indolent cancer, while Gleason patterns 4 and above were considered aggressive cancer. Regions of overlapping Gleason patterns 3 and 4 were considered aggressive cancer.

**FIGURE 1:**
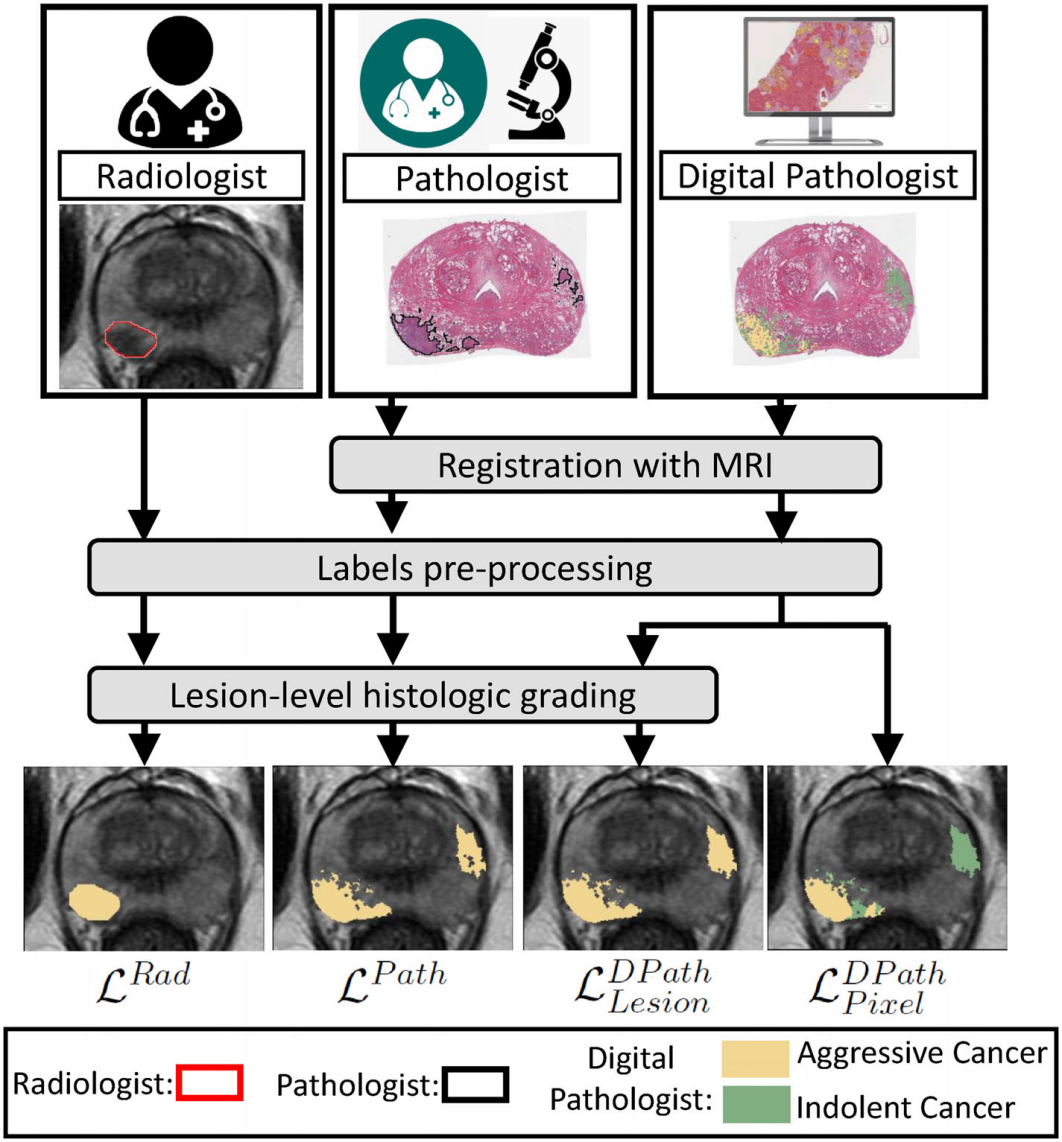
Radiologists, pathologists, or digital pathologists are used to create labels on MRI and serve to train deep learning models to detect cancer and aggressive cancer on MRI. The pathology labels ($L^{\mathrm{Path}}$, $L_{\mathrm{Lesion}}^{\mathrm{DPath}}$, and $L_{\mathrm{Pixel}}^{\mathrm{DPath}}$) are derived through annotations on whole‐mount histopathology images and are mapped onto MRI through MRI‐histopathology registration. The pixel‐level digital pathologist label ($L_{\mathrm{Pixel}}^{\mathrm{DPath}}$) enables identifying aggressive and indolent cancer components in mixed lesions, unlike the other label types

**FIGURE 2:**
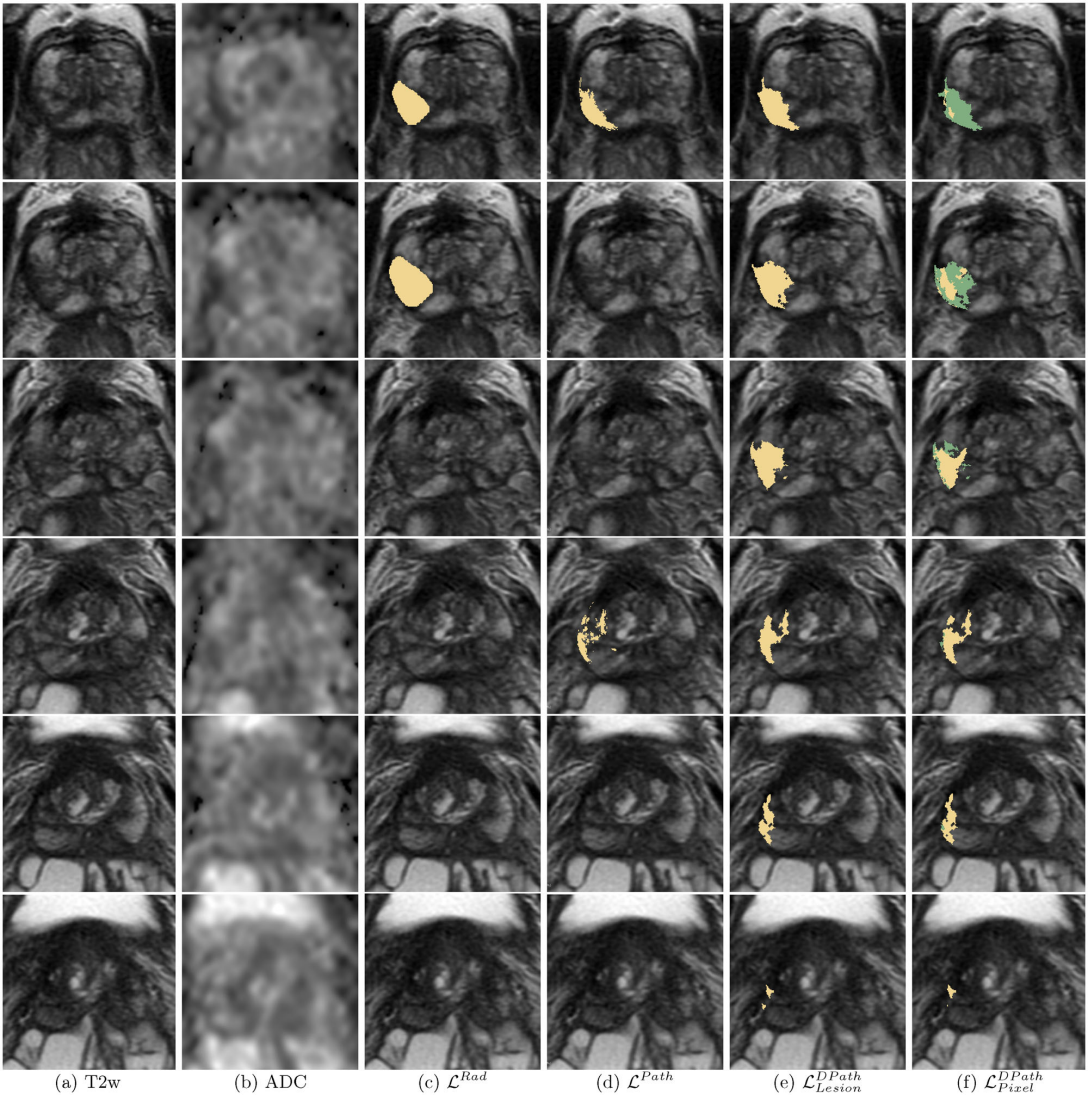
Differences in labeling strategies in a typical patient in cohort C1 test (aggressive cancer—yellow, indolent cancer—green) showed on (a) T2w images and (b) ADC images. The (c) radiologist labels ($L^{\mathrm{Rad}}$) and (d) pathologist labels ($L^{\mathrm{Path}}$) are present on some slices while the (e) lesion‐level digital pathologist labels ($L_{\mathrm{Lesion}}^{\mathrm{DPath}}$), and (f) pixel‐level digital pathologist labels ($L_{\mathrm{Pixel}}^{\mathrm{DPath}}$) exist on all slices. Digital pathologist labels strongly agree with pathologists while annotating aggressive and indolent cancer components in mixed lesions

Figure 1 shows the flowchart for obtaining the different label types, described below:

1. $L^{\mathrm{Rad}}$: Experienced radiologists outlined suspicious lesions on MR images prior to biopsy, and assigned PI‐RADS scores to each lesion as part of routine clinical care. These radiologist‐annotated lesions with PI‐RADS scores ≥3, after pathology confirmation were considered as $L^{\mathrm{Rad}}$ labels (Figure 2c). The site of the lesion suspicious for cancer was outlined on each MRI by one experienced radiologist from the team of eleven board‐certified radiologists in our institution, experience ranging between one and forty years of post‐residency, median years).
  Whole‐mount histopathology specimens and histologic grade labels predicted by the digital pathologist^25^ on these specimens were used to confirm whether lesions outlined by radiologists corresponded to aggressive cancer (see “pathology confirmation of radiologist labels” below). The pixel‐level Gleason patterns or histologic grade labels on histopathology images^25^ predicted by the digital pathologist were mapped onto pre‐operative MRI using an MRI‐histopathology registration^31^ platform (see Section 2.3). The digital pathologist predictions inside each radiologist annotation were used to derive pathology confirmations for that lesion. If a radiologist outline contained at least 1% digital pathologist‐predicted aggressive pixels, the annotation was considered as an aggressive lesion. If the radiologist outline had less than 1% aggressive pixels, but had at least 1% digital pathologist‐predicted indolent pixels, it was considered as an indolent lesion. If a radiologist outline had less than 1% aggressive or indolent pixels, it was considered as benign tissue.
  The 1% of lesion volume threshold for labeling lesions as aggressive or indolent was decided based on the resampled and registered MRI and histopathology volumes (i.e., *X*–*Y* size of 224 × 224, with pixel sizes of 0.29 × 0.29 mm^2^). This 1% threshold was selected to ensure that aggressive cancer was not missed. Our prior study^21^ had experimented with the threshold value for defining aggressive lesions for cohort C1, and 1% being the more stringent threshold for aggressive cancers was chosen in this study.
2. $L^{\mathrm{Path}}$: An expert pathologist (C.A.K. with >10 years of experience) outlined the extent of cancer on whole‐mount histopathology images. These pathologist annotations were converted to 3D lesions using morphological processing (see Section 2.3). The digital pathologist‐derived Gleason patterns^25^ were used to label each pathologist‐annotated lesion into aggressive or indolent, in a way similar to the radiologist labels (at least 1% aggressive pixels within the pathologist outline to be considered as an aggressive lesion). The pathologist labels were mapped onto pre‐operative MRI using the MRI‐histopathology registration platform^31^ (Figure 2d).
3. $L_{\mathrm{Lesion}}^{\mathrm{DPath}}$: The pixel‐level histologic grade labels from the digital pathologist were converted into lesion‐level annotations through morphological processing (see Section 2.3) and by considering the percentage of aggressive cancer pixels within a lesion outline, in a way similar to $L^{\mathrm{Rad}}$ and $L^{\mathrm{Path}}$. These lesion‐level digital pathologist labels were then mapped onto MRI using the MRI‐histopathology registration platform^31^ (Figure 2e).
4. $L_{\mathrm{Pixel}}^{\mathrm{DPath}}$: The pixel‐level histologic grade labels from the digital pathologist was used to derive pixel‐level aggressive and indolent labels for the entire prostate (Figure 2f). Unlike any other label type, pixel‐level digital pathologist labels $L_{\mathrm{Pixel}}^{\mathrm{DPath}}$ selectively labeled aggressive and indolent components of mixed lesions, instead of labeling the entire lesion as aggressive or indolent.

**Pathology confirmation of radiologist labels**:

Our study relied on the digital pathologist^25^ aggressive and indolent labels on whole‐mount histopathology images to provide pathology confirmation and type for the radiologist lesions in cohort C1. Other prior studies^41^ have used histopathology information from targeted biopsy, yet we preferred the more accurate approach of using whole‐mount images for pathology confirmation. Moreover, some of our patients lacked targeted biopsy information (i.e., they had systematic biopsy without lesion targeting or biopsies at outside institutions), further motivating the use of whole‐mount histopathology images for pathology confirmation. Although pathologist‐assigned cancer outlines were available on whole‐mount histopathology images for all patients in cohort C1, pathologist‐assigned Gleason grade labels were unavailable. It is tedious and impractically time consuming for pathologists to assign Gleason grade groups to each lesion on the whole‐mount histopathology images. As such, digital pathologist annotations of Gleason grades provided a reliable, time‐ and labor‐efficient approach for pathology confirmation of radiologist labels.

The digital pathologist used in this study was trained and validated using 1133 prostate needle biopsies on a patient population from a different institution following different scanning protocols than our institution.^25^ When validated on 700 patients and compared with reference standards from three certified pathologists, the digital pathologist showed high diagnostic concordance (κ = 0.907) between predicted grade group and the reference standard, and a high correlation coefficient of 0.97 between tumor length measurements predicted by the digital pathologist and the reference standard.^25^ An independent study from our team^42^ performed external validation of the digital pathologist model^25^ on 500 (1 mm^2^) tiles from 150 whole‐mount prostatectomy specimens from our institution. Consensus from two experienced uropathologists were used to establish the reference standard, with a third expert to evaluate discordant cases. Despite being trained on prostate needle biopsies from a different institution, the digital pathologist demonstrated high agreement with expert uropathologists from our institution in distinguishing between benign versus cancerous tissue (κ = 0.927) and between low risk (benign, Gleason grade group 1, Gleason grade group 2) versus high risk disease (κ = 0.858) on whole‐mount histopathology images. When these digital pathologist labels are used for labeling MRI lesions as aggressive or indolent, the errors are much reduced due to the lower resolution of MRI with respect to the original high‐resolution histopathology images (0.29 mm × 0.29 mm vs. 0.0005 mm × 0.0005 mm).

In order to study the concordance between pathology confirmation from targeted biopsy and the digital pathologist on whole‐mount histopathology images, we analyzed 69 patients in C1‐train that had both targeted biopsy and digital pathologist confirmations. There were a total of 89 radiologist‐annotated lesions in these 69 patients, and after pathology confirmation these correspond to 67 of the $L^{\mathrm{Rad}}$ labels in cohort C1 train (Table 2). We found that the digital pathologist labels agreed with the targeted biopsy confirmations in 77.5% (69/89) of the lesions. The digital pathologist upgraded 11.2% (10/89) of the lesions (benign on targeted biopsy upgraded to indolent/aggressive cancer by digital pathologist, or indolent cancer on targeted biopsy upgraded to aggressive cancer by digital pathologist), and downgraded 11.2% (10/89) of the lesions (indolent or aggressive on targeted biopsy downgraded to benign by digital pathologist, or aggressive on targeted biopsy downgraded to indolent or benign by digital pathologist). These upgrades could be due to sampling errors on targeted biopsy. Seven of the ten downgraded lesions had small proportions of cancer (<5% cancerous tissue) or aggressive cancer (⩽15% of Gleason pattern 4 or above in the cancerous tissue) in the targeted biopsy specimens, and small lesions (<250 mm^3^ lesion volumes) outlined by pathologist and digital pathologists on whole‐mount histopathology images. The remaining three downgrades were due to MRI‐histopathology registration errors or missing histopathology tissue from the whole‐mount specimens. Nonetheless, the digital pathologist labels provide a standardized approach for pathology confirmation of radiologist annotations in the absence of targeted biopsy information. The use of digital pathologist labels for pathology confirmation of radiologist annotations is also consistent with its use to label pathologist lesions into aggressive or indolent in this study.

**TABLE 2:** Descriptive statistics of annotations from the different label types. Statistics for number of patients with labels are irrespective of lesion volume. Lesions with volume ≥250 mm^3^ were considered in this study, whereas lesions with volume <250 mm^3^ were discarded from this study. The large number of discarded lesions for pathology labels ($L^{\mathrm{Rad}}$, $L_{\mathrm{Lesion}}^{\mathrm{DPath}}$, and $L_{\mathrm{Pixel}}^{\mathrm{DPath}}$) arise due to difference in resolution between prostate whole‐mount histopathology and MR images, and mapping of gland‐level labels from whole‐mount histopathology onto MRI

Cohort	C1 train				C1 test				C2
labels	$L^{\mathrm{Rad}}$	$L^{\mathrm{Path}}$	$L_{\mathrm{Lesion}}^{\mathrm{DPath}}$	$L_{\mathrm{Pixel}}^{\mathrm{DPath}}$	$L^{\mathrm{Rad}}$	$L^{\mathrm{Path}}$	$L_{\mathrm{Lesion}}^{\mathrm{DPath}}$	$L_{\mathrm{Pixel}}^{\mathrm{DPath}}$	$L^{\mathrm{Rad}}$
# of patients	75	75	75	75	40	40	40	40	275
# of patients with cancer	75	75	75	75	40	40	40	40	160
# of patients with labels	71	75	75	75	31	40	40	40	160
**Lesions with volume** ≥ **250 mm** ^3^ *considered* **in analysis**									
# of lesions	76	86	85	82	30	44	43	43	193
# of aggressive lesions	63	80	83	49	25	40	42	31	132
# of indolent lesions	13	5	3	33	5	4	1	12	61
Lesion volume (${\text{mm}}^{3}$)									
Mean	2073	2599	2334	2170	1683	2463	2516	2203	1632
Std	3353	4603	3865	3778	1406	2816	2674	2589	2079
Median	1071	1191	1105	916	1118	1334	1522	1262	
**Lesions with volume** < **250 mm** ^3^ *discarded* **from the analysis**									
# of lesions	4	1117	4720	4720	3	493	2489	2489	0
Lesion volume (${\text{mm}}^{3}$)									
Mean	171	9	3	3	117	11	3	3	N/A
Std	44	26	13	13	56	30	16	16	N/A
Median	174	1.4	0.4	0.4	87.9	1.3	0.4	0.4	N/A

**Cohort C2**: Patients in cohort C2 only had pathology‐confirmed radiologist labels $L^{\mathrm{Rad}}$. Since all patients in cohort C2 had targeted biopsy at our institution, pathology confirmation for the radiologist annotations in cohort C2 were derived from pathology of targeted biopsies. Radiologist lesions with targeted biopsy Gleason grade group ≥2 were considered as aggressive lesions, whereas lesions with targeted biopsy Gleason grade group of 1 were considered indolent lesions. Radiologist‐annotated lesions whose targeted biopsies were benign, were considered as normal tissue. Table 2 details the number of aggressive, indolent, and cancerous lesions with their mean volumes annotated by each label type in both cohorts.

#### 2.2.2 Prostate segmentations

Prostate gland segmentations were available on all T2w MRI slices for all patients in both cohorts. In addition, prostate gland segmentations were also available on all histopathology images of cohort C1. Prostate segmentations on all T2w slices were initially performed by medical students and trainees (with 6+ months experience in this task) and were carefully reviewed by our experts (C.A.K.—a pathologist with 14 years experience, G.A.S.—a urologic oncologist with 13 years of experience, P.G.—a body MR imaging radiologist with 14 years of experience, and M.R.—an image analytics expert with 10 years of experience working on prostate cancer).

### 2.3 Data preprocessing

#### 2.3.1 MRI and histopathology images

The data preprocessing was similar to our prior studies,^21^, ^26^ including (1) registration of the pre‐operative MRI and post‐operative histopathology images using the RAPSODI registration platform^31^ for cohort C1 (see Section II.A. in the Supporting Information), (2) manual affine registrations between T2w and ADC images for cohort C1, (3) cropping and resampling to have the same pixel size (0.29 mm × 0.29 mm) and the same *X*–*Y* dimensions (224 × 224) for both cohorts (see Section II.B. in the Supporting Information), (4) MRI intensity standardization^43^, ^44^ and normalization for both cohorts (see Section II.C. in the Supporting Information).

#### 2.3.2 Labels

The label preprocessing steps included forming lesions continuous in the MRI volume from pixel‐level annotations using morphological closing and connected component analysis. The morphological closing operation was performed using a 3D structuring element formed by stacking 3 disks of sizes 0.5, 1.5, and 0.5 mm. This structuring element was chosen to ensure that the generated lesions from pixel‐level annotations faithfully represented the original annotations.

#### 2.3.3 Discarded Lesions

**FIGURE 3:**
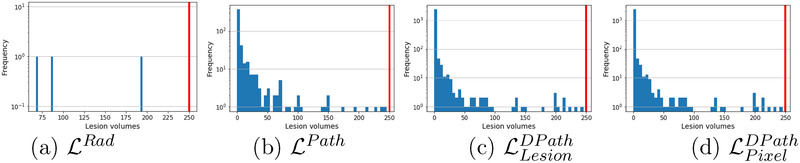
Distribution of lesion volumes of discarded lesions for (a) radiologist ($L^{\mathrm{Rad}}$), (b) pathologist ($L^{\mathrm{Path}}$), (c) lesion‐level digital pathologist $\left(L_{\mathrm{Lesion}}^{\mathrm{DPath}}\right)$, and pixel‐level digital pathologist ($L_{\mathrm{Pixel}}^{\mathrm{DPath}}$) labels for cohort C1 test. The red vertical line indicates the threshold lesion volume of 250 mm^3^. Only three radiologist lesions in C1 test were discarded, whereas, a large number of pathology lesions with predominantly tiny lesion volumes (median discarded lesion volume 0.4–1.3 mm^3^) were discarded. The *y*‐axis shows the frequency of distribution in log scale, while the *x*‐axis shows the lesion volume in mm^3^

**FIGURE 4:**
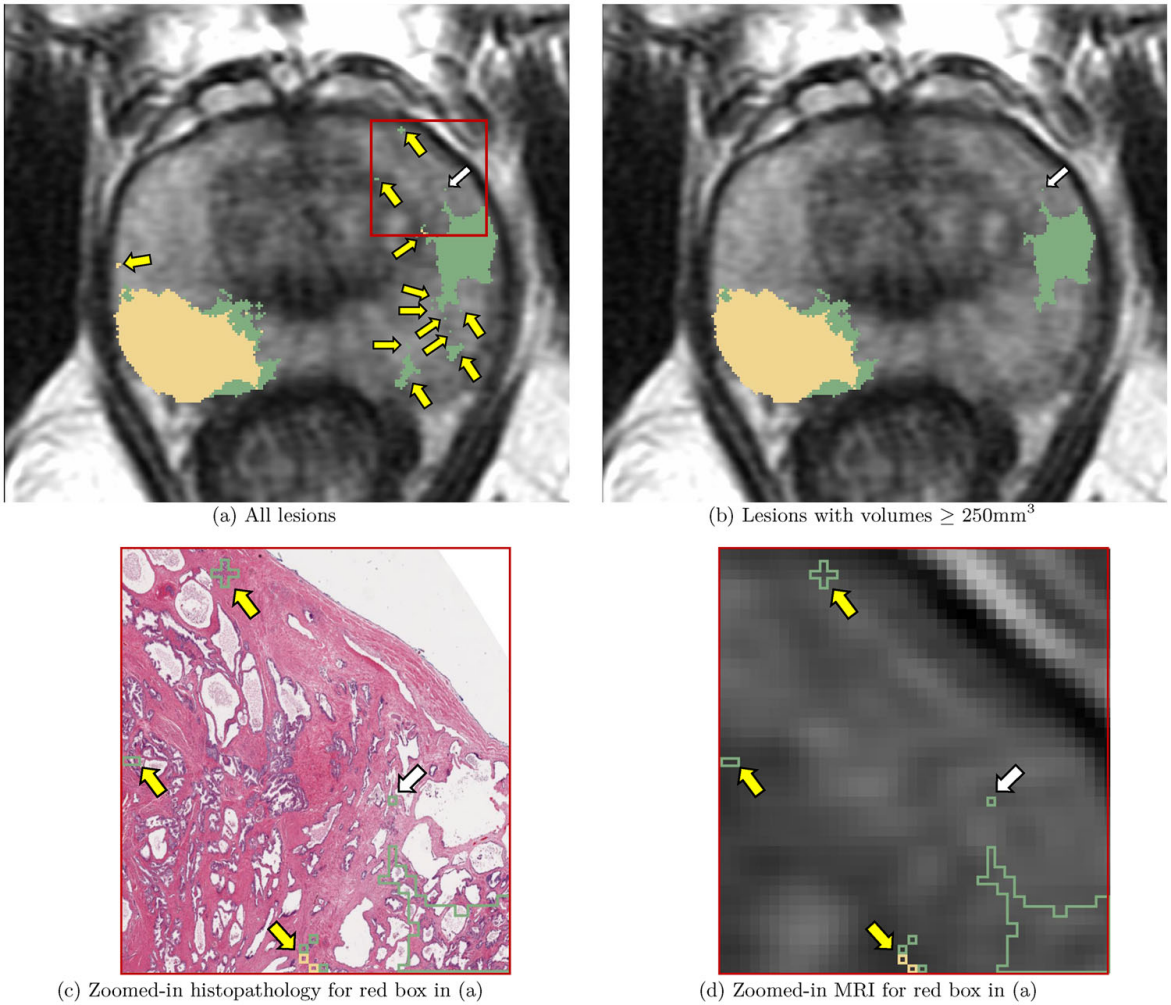
The difference in resolution between the whole‐mount histopathology and the MR images, and the detailed gland‐level annotations of pathology labels, often result in tiny lesions which are (a) only a few pixels on MRI and clinically insignificant (shown by yellow arrows). Discarding small lesions with volumes <250 mm^3^ result in (b) cleaner and clinically meaningful lesions for training and evaluation of digital radiologist models. Zooming into these tiny lesions (red box in (a)) on (c) high resolution histopathology and (d) the registered MRI further reveals these are not clinically meaningful to be detected on MRI. While tiny, the lesion shown by the white arrow is not discarded as it gets connected to the lesion visible in the subsequent MRI slices

Lesion volume (LV) was computed on pre‐processed MRI volume using the following formula:

$$\text{LV}={\text{PS}}_{x}\times {\text{PS}}_{y}\times D_{z}\times N_{L}$$

where, ${\text{PS}}_{x}$ and ${\text{PS}}_{y}$ denotes the MR image pixel sizes in the *X*–*Y* dimensions (0.29 mm each), $D_{z}$ denotes the distance between two consecutive slices (3–4.2 mm), and $N_{L}$ denotes the number of pixels in the 3D lesion after morphological closing and connected component analysis. Lesions with a volume less than 250 mm^3^ were discarded from this study as these smaller lesions (≈6 mm × 6 mm × 6 mm) are unlikely to be seen on MRI, and have been considered as clinically insignificant in prior studies.^21^, ^26^, ^45^ Moreover, according to the PI‐RADS v2 guidelines,^46^ a prostate lesion is considered to be clinically significant cancer only if it has a lesion volume ≥500 mm^3^. We were more conservative than the PI‐RADS v2 guidelines and used half of the 500 mm^3^ threshold to discard lesions from training and evaluation of machine learning models. Analyzing the discarded lesions and the distribution of their lesion volumes in cohort C1 (Table 2 and Figure 3), we note that the pathology labels ($L^{\mathrm{Path}}$, $L_{\mathrm{Lesion}}^{\mathrm{DPath}}$, $L_{\mathrm{Pixel}}^{\mathrm{DPath}}$) have a large number of discarded lesions with median lesion volumes ranging from 0.4 mm^3^ (≈0.67 mm × 0.67 mm × 0.67 mm) to 1.4 mm^3^ (≈1.1 mm × 1.1 mm × 1.1 mm). Such tiny lesions when mapped onto MRI occupy only a few pixels on a single MRI slice, and are invisible or hardly visible to radiologists attempting to interpret the MR image. Thereby lesions with only a few pixels on MRI are not considered clinically relevant (Figure 4) and are not the aim of our study. Such a large number of tiny lesions for pathology labels occur due to the difference in resolution between the whole‐mount histopathology images and the MR images, and the gland‐level detailed annotation of the pathology labels on histopathology images. In order to have clean, meaningful labels, it is essential to filter out these tiny lesions, both during training and evaluation of the digital radiologists.

### 2.4 Model architectures

**FIGURE 5:**
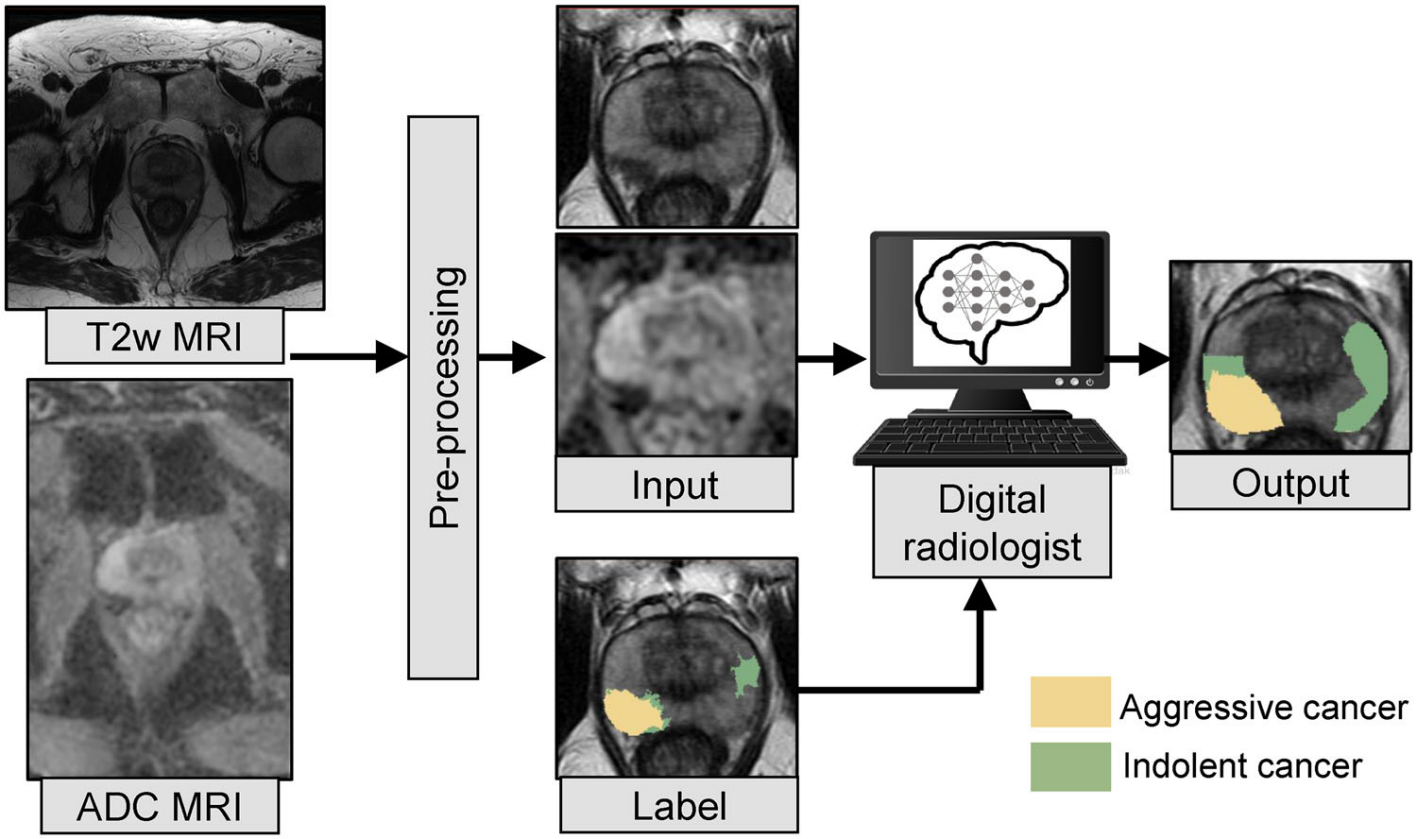
All the digital radiologist models (SPCNet, U‐Net, branched U‐Net, and DeepLabv3+) are trained with T2w and ADC images of the prostate as inputs. Each model is trained with one of the four label types as ground truth at a time. The DeepLabv3+ model is trained in a 2D fashion, with a single slice of T2w and ADC image as input (as shown in this figure), while the other models are trained in a 2.5D fashion with three consecutive MRI slices as inputs. Pre‐processing of the T2w and ADC images includes registration, cropping, and resampling around the prostate, and MRI intensity standardization and normalization

Four different deep learning model architectures (SPCNet,^21^ U‐Net,^19^, ^22^, ^37^, ^47^ branched U‐Net, and DeepLabv3+^18^) were trained using each of the four label types. These four deep learning models were selected based on their previous performance in detecting and localizing prostate cancer (details of these architectures in Section III of the Supporting Information). All model architectures were evaluated to assess whether the effects of different labeling strategies were independent of the model architecture used. Each model takes in T2w and ADC images of the prostate as inputs, and using one of the four label types as ground truth, trains a digital radiologist model to detect, localize, and selectively identify aggressive and indolent cancer (Figure 5). Three consecutive slices of T2w‐MRI and ADC images were used as inputs to all models, except for DeepLabv3+ which takes in a single slice of T2w and ADC images as input. All models were trained using a class‐balanced cross‐entropy loss function to enable multi‐class prediction of each prostate pixel into one of the three classes: normal tissue, indolent cancer, and aggressive cancer. A softmax activation function was used in the last layer of each model, and each prostate pixel was assigned the class with the maximum predicted probability. All models were trained in a five‐fold cross‐validation setting. No post‐processing was done on the predicted labels.

### 2.5 Experimental design

The experimental design was setup to study the following:

#### 2.5.1 Comparison between labeling strategies

The different labels ($L^{\mathrm{Rad}}$, $L^{\mathrm{Path}}$, $L_{\mathrm{Lesion}}^{\mathrm{DPath}}$, and $L_{\mathrm{Pixel}}^{\mathrm{DPath}}$) in cohort C1 test were analyzed with respect to each other in detecting and localizing cancer and aggressive cancer. This analysis was done to study the concordance between the labels themselves, without any digital radiologist training.

#### 2.5.2 Establishing the best digital radiologist architecture

Four different deep learning model architectures (SPCNet, U‐Net, branched U‐Net, and DeepLabv3+) were trained on C1‐train, each with the four different label types ($L^{\mathrm{Rad}}$, $L^{\mathrm{Path}}$, $L_{\mathrm{Lesion}}^{\mathrm{DPath}}$, and $L_{\mathrm{Pixel}}^{\mathrm{DPath}}$), resulting in 16 different digital radiologists. Each model was trained in exactly the same way, with the same pre‐processed data, class‐balanced cross‐entropy loss, batch size of 22, Adam optimizer, and 30 training epochs. A learning rate of 10^−4^ was used for SPCNet and branched U‐Net, 10^−5^ was used for U‐Net, and 10^−3^ was used for DeepLabv3+ architectures. These learning rates were chosen based on optimum performance in the validation set over a range of learning rates ($1\times 10^{-5}$, $3\times 10^{-5}$, $1\times 10^{-4}$, $3\times 10^{-4}$, $1\times 10^{-3}$, $3\times 10^{-3}$, $1\times 10^{-2}$, and $3\times 10^{-2}$). The 16 different digital radiologist models were evaluated for the tasks of detecting cancer and aggressive cancer in cohorts C1 test, and in detecting cancer, aggressive cancer, and indolent cancer in cohort C2. The best digital radiologist model architecture was then chosen from the four different architectures (SPCNet, U‐Net, branched U‐Net, and DeepLabv3+) based on their comparative evaluation.

#### 2.5.3 Studying the effect of different labeling strategies on digital radiologist performance

The effect of the different label types on the performance of the digital radiologist they train was then studied by analyzing the performance of the different digital radiologist model architectures. It was also studied whether the effect of the label types on digital radiologist performance was broadly applicable to any digital radiologist model architecture.

### 2.6 Evaluation methods

The trained digital radiologist models were evaluated in cohort C1‐test with respect to all four label types ($L^{\mathrm{Rad}}$, $L^{\mathrm{Path}}$, $L_{\mathrm{Lesion}}^{\mathrm{DPath}}$, and $L_{\mathrm{Pixel}}^{\mathrm{DPath}}$). Evaluation in cohort C1 test generated 4 × 4 matrices for each evaluation metric, showing how a digital radiologist trained with one label type performed when evaluated with all the other label types. The trained digital radiologist models were also evaluated in cohort C2, which only had pathology‐confirmed radiologist labels ($L^{\mathrm{Rad}}$). Evaluation in cohort C2 enabled studying generalizability of digital radiologists trained with different label types in an independent test set with different distribution of prostate cancer than cohort C1.

The digital radiologists were evaluated for their ability to detect and localize cancer (combined aggressive and indolent subtypes), aggressive cancer, and indolent cancer on prostate MRI on a lesion level. For the lesion‐level evaluation, a sextant‐based approach was used^21^, ^26^ (detailed in Section IV of the Supporting information). True positives and false negatives were assessed using the ground truth and predicted labels, whereas true negatives and false positives were assessed by splitting the prostate into sextants, by first dividing it into left and right halves, and then dividing each half into three roughly equal regions (base, mid, and apex) along the *Z*‐axis (Figure S1). This sextant‐based lesion‐level evaluation is based upon how prostate biopsies are done in clinical practice, with two systematic biopsy cores from each sextant and additional targeted biopsies directed at the lesions. All evaluation was performed on a per‐patient basis, and mean and standard deviation numbers for the entire test sets were reported. Lesion‐level ROC‐AUC, sensitivity, specificity, and Dice coefficients were used as evaluation metrics (details of evaluation metrics reported in Section V of the Supporting Information).

## 3 RESULTS

Our comparison of different MR image‐labeling approaches consisted of three parts. First, we compared the different labeling schemes to evaluate the accuracy of the radiologist and digital pathologist labels relative to the pathologist labels, irrespective of digital radiologist training. Second, we compared multiple deep learning architectures (i.e., different digital radiologist models) to identify the one that performed best on the task of detecting prostate cancer and aggressive prostate cancer on MRI. Third, we carried out a thorough analysis of the performance of all the deep learning architectures (digital radiologist models) in the context of the different labeling strategies.

### 3.1 Comparison between labeling strategies

Annotating cancer extent on radiology or pathology images is tedious and rarely required for routine clinical care. Thus, for all practical purposes, for each patient, clinicians often outline cancerous lesions in some slices, for example, slice with the larger extent, and skip the same lesion when it continues in other slices. Moreover, while radiologists and pathologists may outline the same lesions, they annotate the extent of the cancer differently. For example, the radiologist‐annotated cancer on two slices (slices 1, 2 in Figure 2c), while the pathologist outlined cancer on slices 1 and 4 (Figure 2d) and skipped slices 2 and 3 due to time constraints and not because there are cancer free. Unlike the radiologist and pathologist labels, the digital pathologist labels exist for all slices (Figure 2e,f), and the pixel‐level digital pathologist label ($L_{\mathrm{Pixel}}^{\mathrm{DPath}}$) selectively identifies the aggressive (yellow) and indolent (green) cancer components in the mixed lesion. While differences exist between pathologist and digital pathologist labels, there is a strong agreement in cancer location and extent (Figures 2 and 7).

**FIGURE 6:**
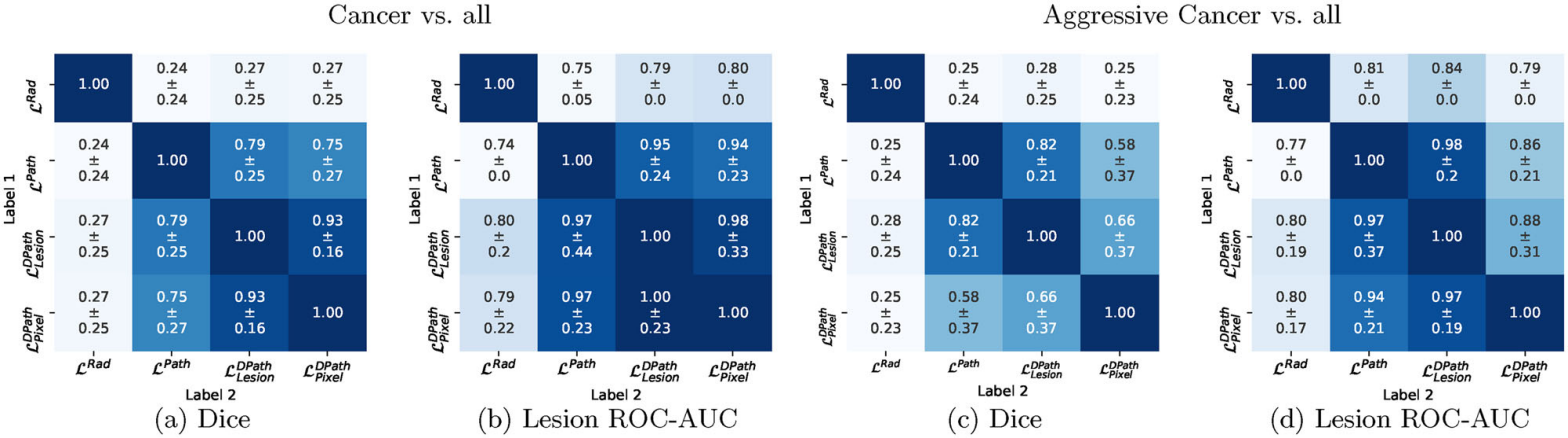
Quantitative comparison between cancer outlines of the different label types. (a) Dice overlap for cancer, (b) lesion‐level ROC‐AUC for cancer, (c) Dice overlap for aggressive cancer, (d) lesion‐level ROC‐AUC for aggressive cancer

**FIGURE 7:**
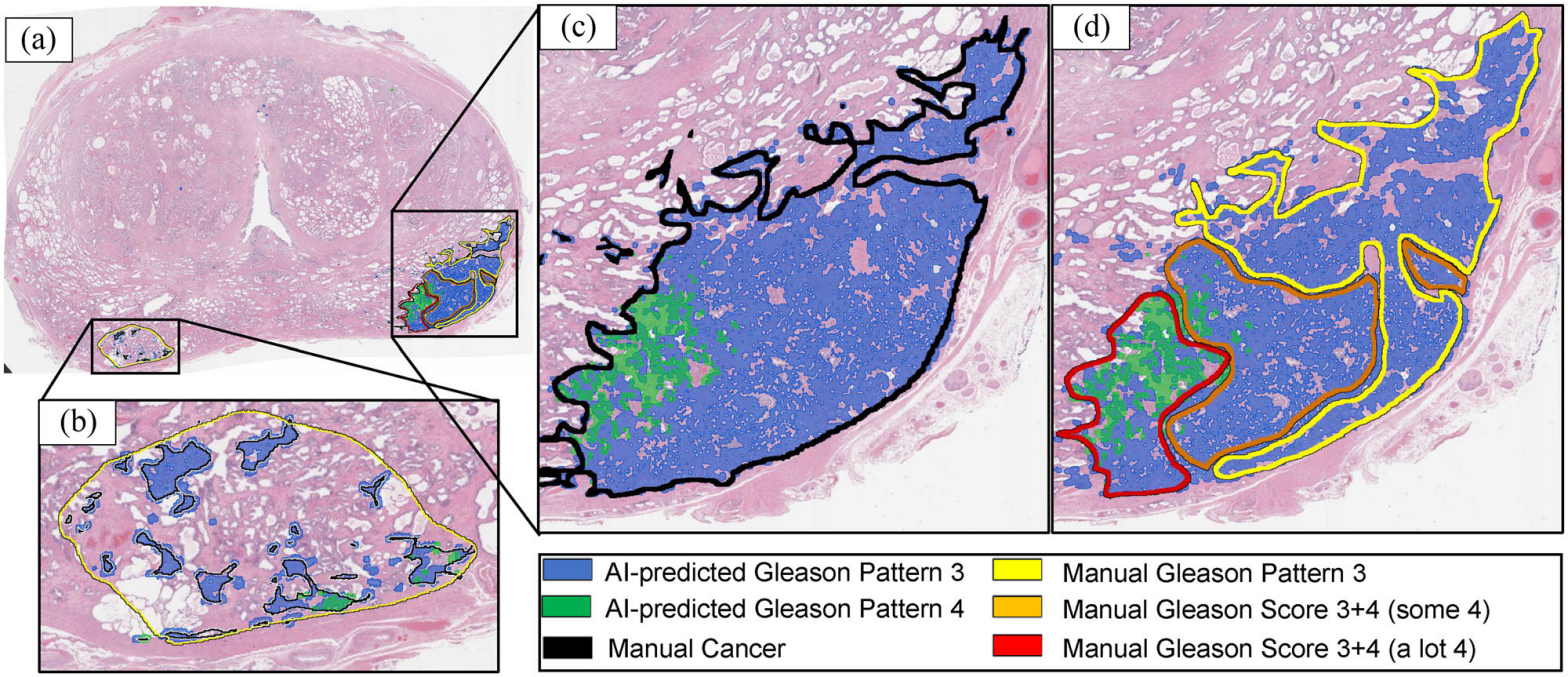
The digital pathologist‐predicted^25^ automated aggressive (Gleason pattern 4, green) and indolent (Gleason pattern 3, blue) cancers visually match the manual cancer annotations by the expert pathologist (black, yellow, orange, and red). (a) Whole‐mount histopathology image with (b–d) close‐up into the two cancer lesions. (C) Cancer labels manually outlined by the expert pathologist (black outline) shows high agreement with overall cancer (combined blue and green) predicted by the digital pathologist model. (b, d) It is impractically time consuming for a human pathologist to manually assign pixel‐level Gleason patterns (yellow, orange, and red) to each gland in detail as done by the digital pathologist (blue and green)

We quantitatively compared the label types for subjects in cohort C1 test using Dice similarity coefficient and lesion‐level ROC‐AUC (Figure 6). The radiologist labels ($L^{\mathrm{Rad}}$) measured low Dice overlaps (0.24‐0.28) and had lesion‐level ROC‐AUCs ranging from 0.75 to 0.84 in cancer and aggressive cancer detection relative to pathology labels ($L^{\mathrm{Path}}$, $L_{\mathrm{Lesion}}^{\mathrm{DPath}}$, and $L_{\mathrm{Pixel}}^{\mathrm{DPath}}$). These lower metrics of radiologist labels can be attributed to radiologists (1) not annotating cancer on all MRI slices, (2) underestimating cancer extents, and (3) missing MRI‐invisible or hardly visible lesions. Radiologist labels have lower lesion volumes than any kind of pathology labels, corresponding to ~68% of the mean $L^{\mathrm{Path}}$ lesion volumes, and ~67% of the mean $L_{\mathrm{Lesion}}^{\mathrm{DPath}}$ lesion volumes (C1‐test in Table 2). Moreover, 11% of patients did not have any radiologist‐outlined lesions but ended up having clinically significant cancer (Table 2). The radiologist labels were from the initial diagnostic read in the clinical care of the patients, essentially in vacuum, without any pathology information. Although this reflects the real‐world scenario of routine clinical care, this also puts radiologists at an unfair disadvantage when comparing their initial diagnostic reads with post‐operative surgical specimens.

The lesion‐level digital pathologist labels ($L_{\mathrm{Lesion}}^{\mathrm{DPath}}$) achieved high (0.79–0.82) Dice overlap and very high agreement in lesion‐level ROC‐AUCs (cancer ROC‐AUCs: 0.94‐1.00; aggressive cancer ROC‐AUCs: 0.86‐0.97) with pathologist labels ($L^{\mathrm{Path}}$). The deviations from a perfect Dice overlap can be attributed to the difference in resolution between the two kinds of pathologist labels, that is, digital pathologists labeling each gland in detail, while it is tedious and impractical to annotate each gland on the whole‐mount prostate histopathology images in detail by a human pathologist (Figure 7). Despite the difference in the level of detail, the concordance between pathologist and digital pathologist labels in distinguishing between benign versus  cancer tissues, as well as indolent versus aggressive cancer is evident from Figure 7. Moreover, the pathologist may have not provided labels on all slices.

The pixel‐level digital pathologist labels ($L_{\mathrm{Pixel}}^{\mathrm{DPath}}$) achieved high Dice overlaps with $L^{\mathrm{Path}}$ and $L_{\mathrm{Lesion}}^{\mathrm{DPath}}$ for cancer, and achieved lower Dice overlaps (0.58 ± 0.37, 0.66 ± 0.37,) with $L^{\mathrm{Path}}$ and $L_{\mathrm{Lesion}}^{\mathrm{DPath}}$ for aggressive cancer. This low aggressive cancer Dice coefficient for $L_{\mathrm{Pixel}}^{\mathrm{DPath}}$ is due to its selective labeling of aggressive and indolent cancer components in mixed cancerous lesions, unlike the other label types which label the entire lesion as aggressive or indolent.

### 3.2 Establishing the best digital radiologist architecture

We compared the four architectures (SPCNet, U‐Net, branched U‐Net, and DeepLabv3+) trained with different label types in detecting and localizing cancer and aggressive cancer on a lesion level (Table 3). In cohort C1 test, models trained were evaluated with respect to pathologist labels ($L^{\mathrm{Path}}$), while in cohort C2, they were evaluated with respect to biopsy‐confirmed radiologist labels ($L^{\mathrm{Rad}}$). SPCNet outperformed other models in most metrics and most evaluation types.

**TABLE 3:** The SPCNet architecture achieved the best performance in detecting cancer and aggressive cancer in both cohorts irrespective of the label type used for training

Cancer versus all								
Cohort C1 test (*N* = 40, number of lesions = 48). Evaluated against $L^{\mathrm{Path}}$								
	AUC‐ROC				Dice			
Trained with label type	SPCNet	U‐Net	Branched U‐Net	DeepLabv3+	SPCNet	U‐Net	Branched U‐Net	DeepLabv3+
$L^{\mathrm{Rad}}$	0.87 ± 0.22	0.84 ± 0.27	0.77 ± 0.33	0.88 ± 0.21	0.37 ± 0.22	0.37 ± 0.22	0.31 ± 0.22	0.34 ± 0.22
$L^{\mathrm{Path}}$	**0.90** ± **0.22**	0.87 ± 0.25	0.82 ± 0.32	0.86 ± 0.21	**0.39** ± **0.19**	0.38 ± 0.22	0.29 ± 0.20	0.32 ± 0.23
$L_{\mathrm{Lesion}}^{\mathrm{DPath}}$	**0.92** ± **0.18**	0.86 ± 0.24	0.89 ± 0.24	0.89 ± 0.19	**0.34** ± **0.2**	0.38 ± 0.23	0.28 ± 0.20	0.32 ± 0.21
$L_{\mathrm{Pixel}}^{\mathrm{DPath}}$	**0.91** ± **0.19**	0.83 ± 0.30	0.83 ± 0.27	**0.91** ± **0.17**	**0.30** ± **0.21**	0.26 ± 0.21	0.25 ± 0.20	**0.30** ± **0.24**
Cohort C2 (*N* = 160, number of lesions = 193). Evaluated against $L^{\mathrm{Rad}}$								
	AUC‐ROC				Dice			
Trained with label type	SPCNet	U‐Net	Branched U‐Net	DeepLabv3+	SPCNet	U‐Net	Branched U‐Net	DeepLabv3+
$L^{\mathrm{Rad}}$	**0.84** ± **0.29**	0.82 ± 0.31	0.82 ± 0.33	0.81 ± 0.34	0.39 ± 0.28	**0.39** ± **0.26**	0.38 ± 0.26	0.39 ± 0.27
$L^{\mathrm{Path}}$	**0.81** ± **0.33**	0.80 ± 0.32	0.78 ± 0.34	**0.81** ± **0.32**	**0.37** ± **0.27**	0.37 ± 0.25	0.36 ± 0.25	0.35 ± 0.25
$L_{\mathrm{Lesion}}^{\mathrm{DPath}}$	**0.81** ± **0.32**	0.78 ± 0.35	0.77 ± 0.35	0.79 ± 0.33	**0.37** ± **0.27**	0.36 ± 0.25	0.35 ± 0.26	0.34 ± 0.25
$L_{\mathrm{Pixel}}^{\mathrm{DPath}}$	0.81 ± 0.31	**0.82** ± **0.32**	0.75 ± 0.36	0.80 ± 0.33	**0.35** ± **0.29**	0.31 ± 0.26	0.33 ± 0.25	0.31 ± 0.26
**Aggressive cancer versus all**								
Cohort C1 test (*N* = 40, number of lesions = 44). Evaluated against $L^{\mathrm{Path}}$								
	AUC‐ROC				Dice			
Trained with label type	SPCNet	U‐Net	Branched U‐Net	DeepLabv3+	SPCNet	U‐Net	Branched U‐Net	DeepLabv3+
$L^{\mathrm{Rad}}$	0.88 ± 0.24	0.87 ± 0.26	0.78 ± 0.32	**0.91** ± **0.20**	**0.36** ± **0.39**	0.36 ± 0.22	0.31 ± 0.22	0.34 ± 0.22
$L^{\mathrm{Path}}$	**0.91** ± **0.21**	0.90 ± 0.24	0.83 ± 0.30	0.90 ± 0.19	**0.39** ± **0.19**	0.38 ± 0.22	0.29 ± 0.20	0.33 ± 0.23
$L_{\mathrm{Lesion}}^{\mathrm{DPath}}$	**0.92** ± **0.19**	0.89 ± 0.22	0.90 ± 0.23	**0.92** ± **0.17**	**0.34** ± **0.20**	0.38 ± 0.23	0.28 ± 0.21	0.33 ± 0.21
$L_{\mathrm{Pixel}}^{\mathrm{DPath}}$	0.91 ± 0.19	0.87 ± 0.28	0.86 ± 0.26	**0.92** ± **0.16**	**0.31** ± **0.21**	0.27 ± 0.21	0.25 ± 0.20	**0.31** ± **0.24**
Cohort C2 (*N* = 160, number of lesions = 132). Evaluated against $L^{\mathrm{Rad}}$								
	AUC‐ROC				Dice			
Trained with Label type	SPCNet	U‐Net	Branched U‐Net	DeepLabv3+	SPCNet	U‐Net	Branched U‐Net	DeepLabv3+
$L^{\mathrm{Rad}}$	**0.89** ± **0.24**	0.72 ± 0.34	0.86 ± 0.30	0.86 ± 0.30	0.43 ± 0.26	0.25 ± 0.19	0.42 ± 0.24	**0.44** ± **0.24**
$L^{\mathrm{Path}}$	**0.87** ± **0.27**	0.67 ± 0.39	0.85 ± 0.30	0.86 ± 0.27	**0.41** ± **0.25**	0.25 ± 0.24	0.40 ± 0.23	0.39 ± 0.24
$L_{\mathrm{Lesion}}^{\mathrm{DPath}}$	**0.87** ± **0.26**	0.70 ± 0.39	0.83 ± 0.23	0.86 ± 0.28	**0.42** ± **0.25**	0.21 ± 0.20	0.39 ± 0.24	0.39 ± 0.25
$L_{\mathrm{Pixel}}^{\mathrm{DPath}}$	**0.88** ± **0.27**	0.79 ± 0.34	0.80 ± 0.33	0.85 ± 0.31	**0.40** ± **0.28**	0.23 ± 0.21	0.36 ± 0.24	0.37 ± 0.26

### 3.3 Studying the effect of different labeling strategies on digital radiologist performance

#### 3.3.1 Qualitative comparison

**FIGURE 8:**
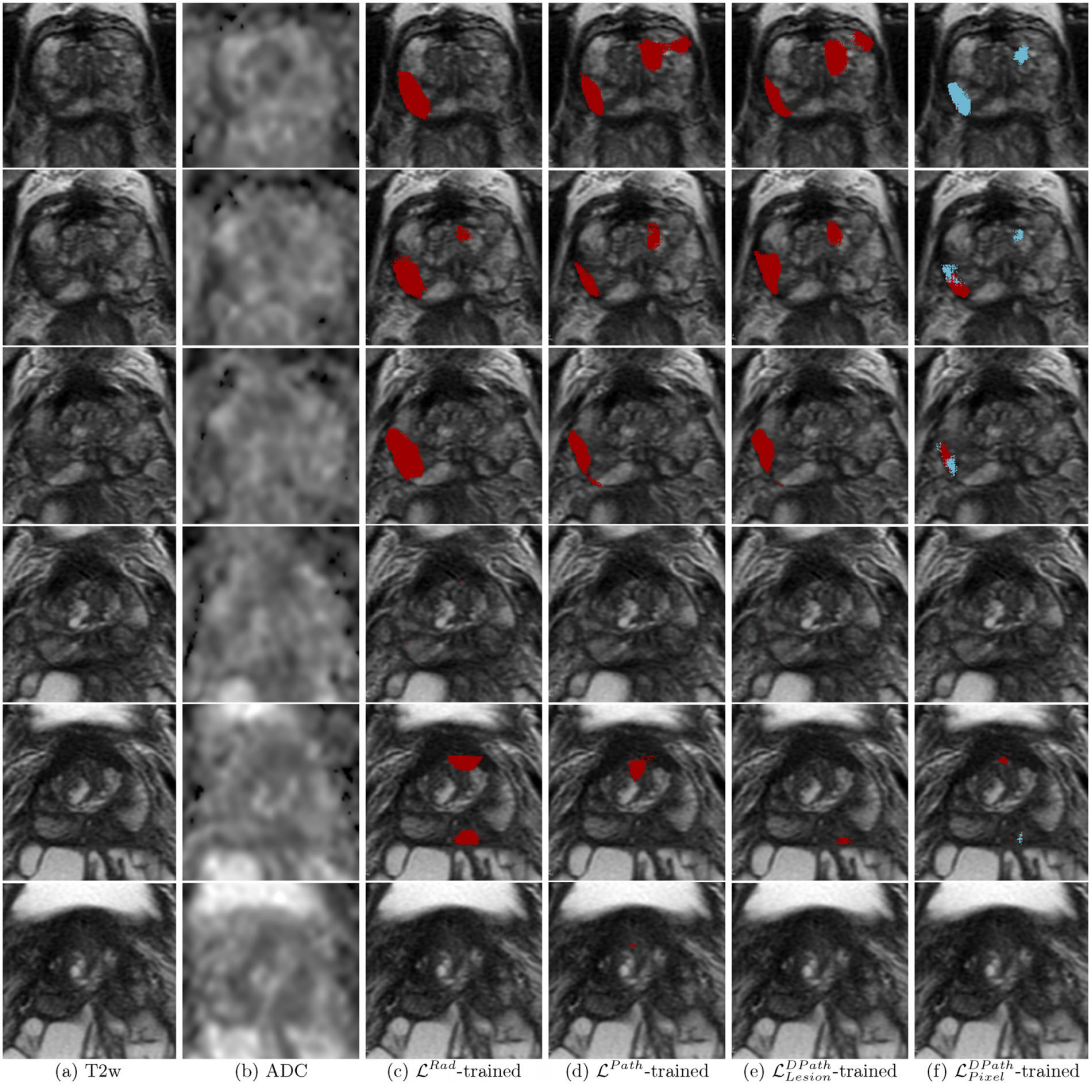
Predictions from SPCNet trained with different label types of a typical patient from cohort C1 test (same as Figure 2) show that only $L_{\mathrm{Pixel}}^{\mathrm{DPath}}$‐trained SPCNet (f) selectively identified the aggressive and indolent cancer components in the lesion, while all other models detected the lesion as aggressive (SPCNet predictions: aggressive cancer [red], indolent cancer [blue[). (a) T2w images, (b) ADC images, (c) $L^{\mathrm{Rad}}$‐trained SPCNet predictions, (d) $L^{\mathrm{Path}}$‐trained SPCNet predictions, (e) $L_{\mathrm{Lesion}}^{\mathrm{DPath}}$‐trained SPCNet predictions, (f) $L_{\mathrm{Pixel}}^{\mathrm{DPath}}$‐trained SPCNet predictions

**FIGURE 9:**
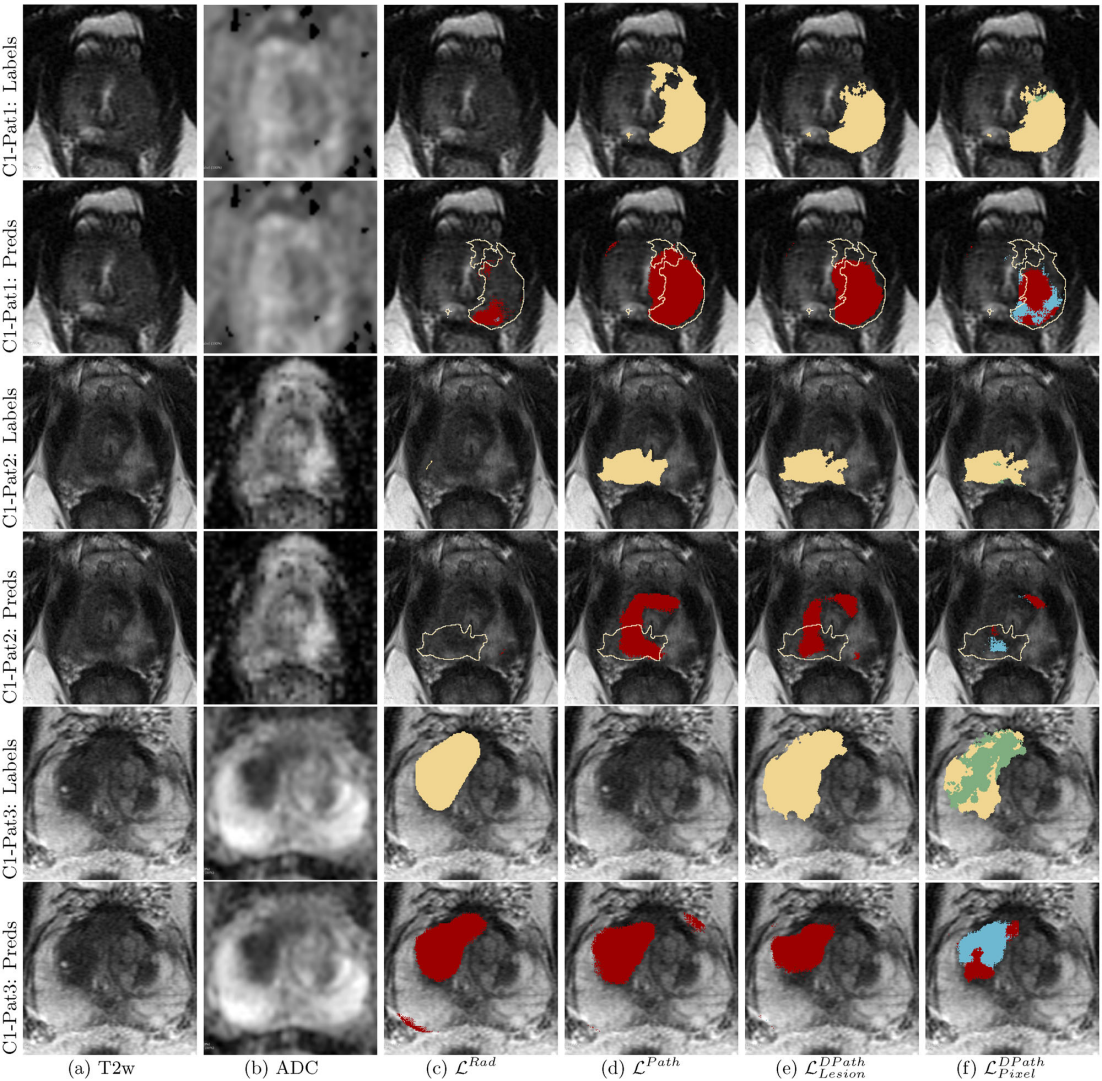
Labels and SPCNet predictions for three different patients from cohort C1 test (labels: aggressive cancer [yellow], indolent cancer [green]); SPCNet predictions: aggressive cancer [red], indolent cancer [blue]) on (a) T2w and (b) ADC images. The (c) $L^{\mathrm{Rad}}$ labels and $L^{\mathrm{Rad}}$‐trained SPCNet predictions may miss cancers or underestimate cancer extent. The (d) $L^{\mathrm{Path}}$ labels and $L^{\mathrm{Path}}$‐trained SPCNet predictions, and the (e) $L_{\mathrm{Lesion}}^{\mathrm{DPath}}$ and $L_{\mathrm{Lesion}}^{\mathrm{DPath}}$‐trained SPCNet predictions show strong agreement in cancer localization and extent. The (f) $L_{\mathrm{Pixel}}^{\mathrm{DPath}}$ and $L_{\mathrm{Pixel}}^{\mathrm{DPath}}$‐trained SPCNet predictions can selectively identify and localize the aggressive and indolent cancer components in the mixed lesions unlike any other label or prediction type. The outline for columns with SPCNet predictions correspond to pathologist annotations. Radiologists and pathologists are not required to annotate cancer extent on all slices of a patient for routine clinical care, but knowing the complete extent of cancer on all slices may be essential to train machine learning models. As such, C1‐Pat3 does not show a $L^{\mathrm{Path}}$ label while cancer is present

**FIGURE 10:**
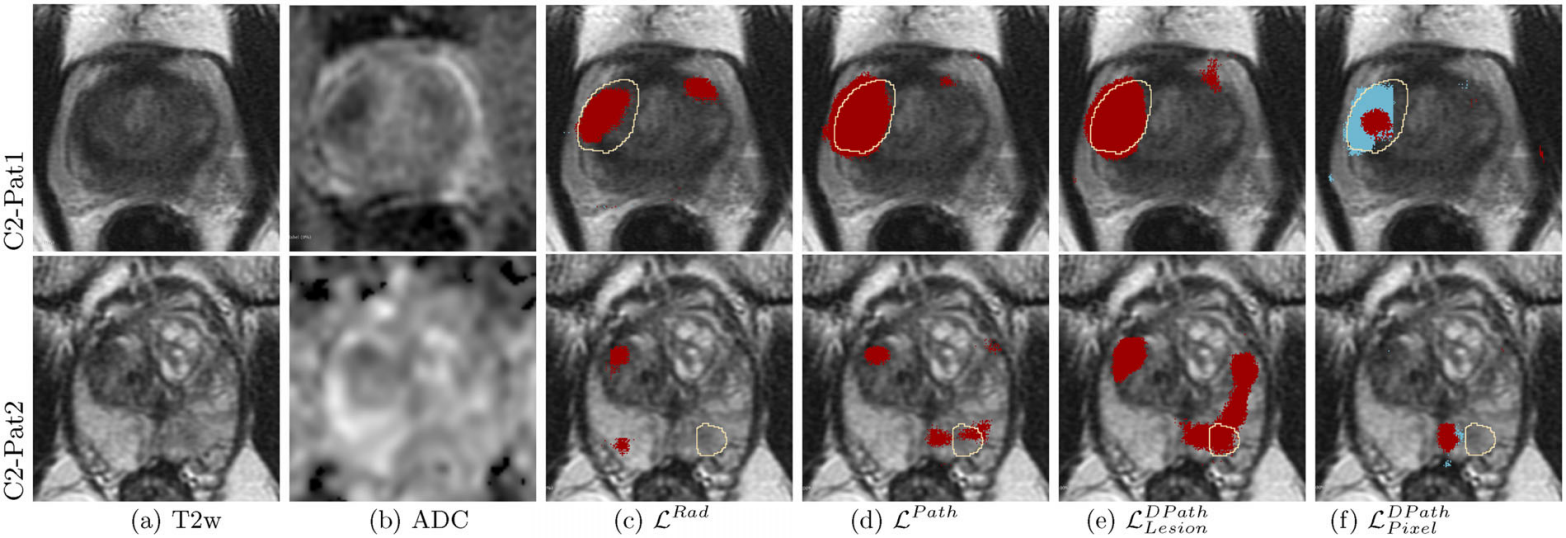
SPCNet predictions for two different patients from cohort C2 on (a) T2w and (b) ADC images. The (c)$L^{\mathrm{Rad}}$‐trained SPCNet predictions miss the cancer in the row 2 patient C2‐Pat2. The (d) $L^{\mathrm{Path}}$‐trained and (e) $L_{\mathrm{Lesion}}^{\mathrm{DPath}}$‐trained SPCNet predictions detect the lesions in both patients, with the (e) $L_{\mathrm{Lesion}}^{\mathrm{DPath}}$‐trained predictions having the highest overlap with the cancer extent. The (f) $L_{\mathrm{Pixel}}^{\mathrm{DPath}}$‐trained SPCNet predictions are slightly off from the $L^{\mathrm{Rad}}$ labels for the row 2 patient C2‐Pat2. The outlines for columns with SPCNet‐predictions correspond to radiologist labels ($L^{\mathrm{Rad}}$)

Digital radiologists trained with radiologist labels ($L^{\mathrm{Rad}})$ could detect cancer in both cohorts (Figures 8, 9, and 10c), but in comparison with other digital radiologists they missed some cancers (Figure 9c, row 4, C1‐Pat2:Preds, and Figure 10c, row 2, C2‐Pat2), and underestimated cancer extent in some patients (Figure 9c, row2, C1‐Pat1:Preds and Figure 10c, row 2, C2‐Pat1).

Digital radiologists trained with lesion‐level pathology labels ($L^{\mathrm{Path}}$ and $L_{\mathrm{Lesion}}^{\mathrm{DPath}}$) had the best (and very similar) performances in detecting and localizing cancer, and also in capturing the true extent of the cancer (Figures 8, 9, 10, columns d and e). Digital radiologists trained with pixel‐level digital pathologist labels ($L_{\mathrm{Pixel}}^{\mathrm{DPath}}$) are the only ones to selectively identify aggressive and indolent cancer in mixed lesions (Figures 8f and 9f, row 6, C1‐Pat3: Preds), albeit sometimes having less cancer extent than the $L^{\mathrm{Path}}$ and $L_{\mathrm{Lesion}}^{\mathrm{DPath}}$‐trained digital radiologists (Figure 9f, row 4, C1‐Pat2: Preds). Predictions from the $L_{\mathrm{Pixel}}^{\mathrm{DPath}}$‐trained digital radiologist for the row 2 patient (C2‐Pat2) is slightly off from the actual ground truth lesion annotation.

#### 3.3.2 Quantitative comparison

**FIGURE 11:**
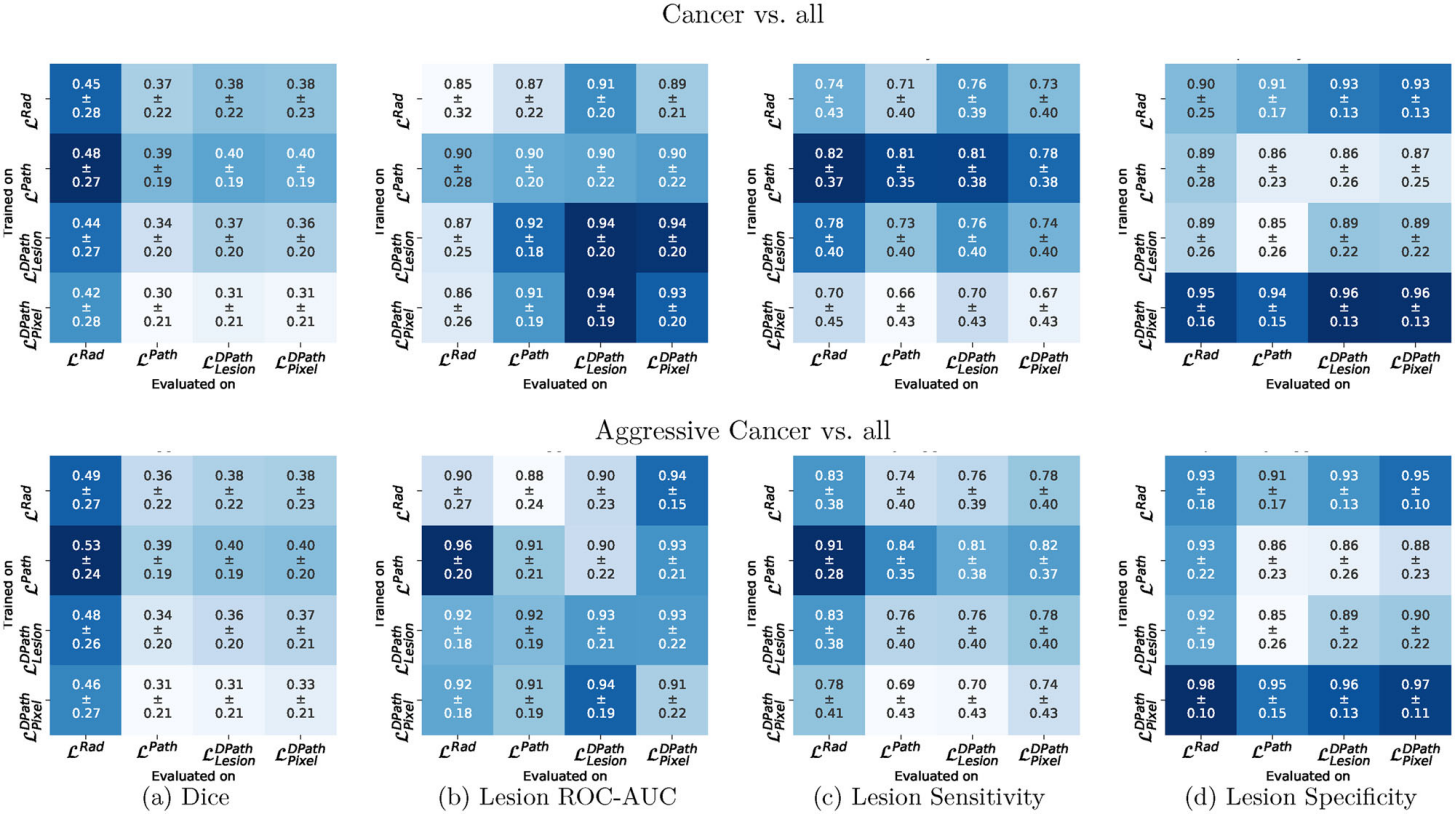
Quantitative comparison between digital radiologist (SPCNet) predictions when trained and evaluated using different label types in cohort C1 test. The top row shows results for cancer detection, while the bottom row shows results for aggressive cancer detection. Darker blue boxes in the 4 × 4 matrices represent higher evaluation metrics.

**Cohort C1 test**: Quantitatively comparing the lesion‐level performance of the digital radiologists trained with the different label types in cohort C1 test showed that the type of label used for training has an effect on digital radiologist performance (Figure 11 and Figures S2–S4). All models trained with each label type were evaluated with respect to all other label types, generating 4 × 4 evaluation matrices for each model and each evaluation metric. A row in the 4 × 4 matrix denotes a model trained with a particular label type, when evaluated with all the label types. A column in the matrix denotes models trained with different label types when evaluated using one particular label type. The 4 × 4 evaluation matrices of the best‐performing digital radiologist model (SPCNet) is presented in this section (Figure 11), whereas evaluation matrices from the other digital radiologist model architectures (U‐Net, branched U‐Net, and DeepLabv3+) are presented in Section V of the Supporting Information (Figures S2–S4). A summary of the performance of all the models (SPCNet, U‐Net, branched U‐Net, and DeepLabv3+) is presented below, when the pathologist labels ($L^{\mathrm{Path}}$) are considered gold standard for evaluation in cohort C1 (second column in each sub‐figure of Figure 11 and Figures S2–S4).

**Models trained with radiologist labels (** $L^{\mathrm{Rad}}$ **)**: The SPCNet, U‐Net, and branched U‐Net models trained with radiologist labels ($L^{\mathrm{Rad}}$) had lower lesion‐level ROC‐AUCs and lower sensitivities than their pathologist label‐trained ($L^{\mathrm{Path}}$) counterparts. The DeepLabv3+ model trained with $L^{\mathrm{Rad}}$ exhibited similar/slightly higher ROC‐AUC and sensitivities when compared to their pathologist label‐trained counterparts. All models trained with $L^{\mathrm{Rad}}$ had lower or very similar Dice to their pathologist label‐trained counterparts.

**Models trained with pathologist labels (** $L^{\mathrm{Path}}$ **)**: The SPCNet and U‐Net models trained with pathologist labels ($L^{\mathrm{Path}}$) achieved the highest ROC‐AUC, Dice coefficient, and sensitivities of all the models. For branched U‐Net and DeepLabv3+ models, the performance of $L^{\mathrm{Path}}$ label‐trained models was similar or closely following the highest performing model.

**Models trained with digital pathologist labels (** $L_{\mathrm{Lesion}}^{\mathrm{DPath}}$, $L_{\mathrm{Pixel}}^{\mathrm{DPath}}$ **)**: All digital radiologist models (SPCNet, U‐Net, branched U‐Net, and DeepLabv3+) when trained with lesion‐level digital pathologist labels ($L_{\mathrm{Lesion}}^{\mathrm{DPath}}$) consistently achieved (a) higher lesion‐level ROC‐AUCs than their radiologist label‐trained ($L^{\mathrm{Rad}}$) counterparts, (b) similar or higher ROC‐AUCs than their pathologist label‐trained ($L^{\mathrm{Path}}$) counterparts, (c) similar or slightly lower Dice compared to their pathologist label‐trained ($L^{\mathrm{Path}}$) counterparts. All models trained with pixel‐level digital pathologist labels ($L_{\mathrm{Pixel}}^{\mathrm{DPath}}$) also exhibited similar ROC‐AUC when compared to their pathologist label‐trained ($L^{\mathrm{Path}}$) counterparts, although they achieved slightly lower Dice coefficients. Most models (SPCNet, U‐Net, and DeepLabv3+) trained with $L_{\mathrm{Pixel}}^{\mathrm{DPath}}$ exhibited the highest specificities. The slightly inferior performance of $L_{\mathrm{Pixel}}^{\mathrm{DPath}}$‐trained models in comparison to $L_{\mathrm{Lesion}}^{\mathrm{DPath}}$‐trained models can be attributed to the nature of these labels identifying indolent and aggressive cancer components on a pixel‐level in mixed lesions, as opposed to the other label types that consider the entire lesion as aggressive or indolent.

The consistent performance of digital radiologists trained with digital pathologist labels ($L_{\mathrm{Lesion}}^{\mathrm{DPath}}$ and $L_{\mathrm{Pixel}}^{\mathrm{DPath}}$) across different model architectures suggest their utility in training digital radiologists, irrespective of the model architecture.

For all digital radiologists, irrespective of model architecture, highest Dice overlaps were achieved when evaluated using radiologist labels ($L^{\mathrm{Rad}}$) (darker blue column 1 of first sub‐figures in Figure 11 and Figures S2–S4). This observation can be attributed to the fact that cancers captured by $L^{\mathrm{Rad}}$ labels are more prominent on MRI, making them easier to be learned by the digital radiologists.

For all digital radiologist model architectures, the performance on the held‐out test sets presented above was obtained by averaging the performances of the five models trained in five‐fold cross‐validation. This approach provides an estimate of the generalizability of models, and also helps deal with bias arising from particular train–test splits. Section VI.B of the Supporting Information includes the performance of the SPCNet‐based digital radiologist model on the validation sets of the five‐folds. Validation set performance also suggests that digital pathologist label‐trained models show consistent and better/similar performance to other label‐trained models across folds.

**Cohort C2**: All models trained with all label types were evaluated only with radiologist labels ($L^{\mathrm{Rad}}$) in cohort C2, as other label types were unavailable for this cohort. Evaluation table for SPCNet (Table 4) is presented below, while evaluation tables for the other models (U‐Net, branched U‐Net, and DeepLabv3+) are presented in Section V of the Supporting information (Tables S2–S4). A summary of the performance of all models in cohort C2 when evaluated with $L^{\mathrm{Rad}}$ labels is presented below.

**TABLE 4:** Lesion‐level evaluation in cohort C2 of the SPCNet models trained using cohort C1 train. Cohort C2 only had biopsy‐confirmed radiologist labels ($L^{\mathrm{Rad}}$), thus all evaluations were with respect to $L^{\mathrm{Rad}}$

Cancer versus all (*N* = 160, number of lesions = 193)				
Trained with label type	AUC‐ROC	Dice	Sens.	Spec.
$L^{\mathrm{Rad}}$	**0.84** ± **0.29**	**0.39** ± **0.28**	0.70 ± 0.42	0.85 ± 0.28
$L^{\mathrm{Path}}$	0.81 ± 0.33	0.37 ± 0.27	0.70 ± 0.43	0.73 ± 0.36
$L_{\mathrm{Lesion}}^{\mathrm{DPath}}$	0.81 ± 0.32	0.37 ± 0.27	**0.71** ± **0.42**	0.78 ± 0.34
$L_{\mathrm{Pixel}}^{\mathrm{DPath}}$	0.81 ± 0.31	0.35 ± 0.29	0.64 ± 0.45	**0.87** ± **0.26**
Aggressive cancer versus all (*N* = 160, number of lesions = 132)				
Trained with label type	AUC‐ROC	Dice	Sens.	Spec.
$L^{\mathrm{Rad}}$	**0.89** ± **0.24**	**0.43** ± **0.26**	0.77 ± 0.39	0.84 ± 0.28
$L^{\mathrm{Path}}$	0.87 ± 0.27	0.41 ± 0.25	0.79 ± 0.39	0.72 ± 0.37
$L_{\mathrm{Lesion}}^{\mathrm{DPath}}$	0.87 ± 0.26	0.42 ± 0.25	**0.81** ± **0.37**	0.77 ± 0.36
$L_{\mathrm{Pixel}}^{\mathrm{DPath}}$	0.88 ± 0.27	0.40 ± 0.28	0.73 ± 0.42	**0.85** ± **0.29**
Indolent cancer versus all (*N* = 160, number of lesions = 61)				
Trained with label type	AUC‐ROC	Dice	Sens.	Spec.
$L^{\mathrm{Rad}}$	0.46 ± 0.42	0.00 ± 0.01	0.02 ± 0.13	0.99 ± 0.01
$L^{\mathrm{Path}}$	0.43 ± 0.43	0.00 ± 0.00	0.00 ± 0.00	**1.00** ± **0.00**
$L_{\mathrm{Lesion}}^{\mathrm{DPath}}$	0.43 ± 0.40	0.00 ± 0.00	0.00 ± 0.00	**1.00** ± **0.00**
$L_{\mathrm{Pixel}}^{\mathrm{DPath}}$	**0.64** ± **0.40**	**0.12** ± **0.17**	**0.33** ± **0.45**	0.94 ± 0.14

**Models trained with radiologist labels (** $L^{\mathrm{Rad}}$ **)**: All models (SPCNet, U‐Net, branched U‐Net, and DeepLabv3+) trained with radiologist labels ($L^{\mathrm{Rad}}$) had the highest lesion‐level ROC‐AUC and Dice overlaps (Table 4 and Tables S2–S4) for cancer and aggressive cancer detection in cohort C2. The better performance of $L^{\mathrm{Rad}}$‐trained models in cohort C2 can be attributed to the fact that evaluation is also with respect to $L^{\mathrm{Rad}}$ in this cohort as other labels are not available.

**Models trained with pathology labels (** $L^{\mathrm{Path}}$ **, LDPath_Lesion, DPath_Pixel)**: For cancer and aggressive cancer detection, all models trained with pathology labels had similar or slightly inferior ROC‐AUC and Dice overlaps in comparison to their radiologist label‐trained counterparts. It may however be noted that using $L^{\mathrm{Rad}}$ as gold standard for evaluation can lead to missed cancers (Section 3.1), which can in turn lead to inferior evaluation metrics for models trained with pathology labels ($L^{\mathrm{Path}}$, $L_{\mathrm{Lesion}}^{\mathrm{DPath}}$, and $L_{\mathrm{Pixel}}^{\mathrm{DPath}}$).

## 4 DISCUSSION

In this study, we performed a detailed analysis to (a) compare different prostate cancer labeling strategies, and (b) study the effects these labeling strategies have on the deep learning models (which we refer to as digital radiologists) that are trained with them. Our qualitative and quantitative evaluations indicate that radiologist labels ($L^{\mathrm{Rad}}$) have lower lesion‐detection rates than pathology labels (labels on whole‐mount histopathology images mapped onto MRI through MRI‐histopathology registration), and do not capture the true extent of cancer, in line with prior studies.^2^, ^4^, ^27^ Subsequently, digital radiologist models trained with $L^{\mathrm{Rad}}$ also have inferior performance when compared to models trained with pathology labels ($L^{\mathrm{Path}}$, $L_{\mathrm{Lesion}}^{\mathrm{DPath}}$, and $L_{\mathrm{Pixel}}^{\mathrm{DPath}}$). Digital pathologist (deep learning method for labeling of Gleason patterns on histopathology images^25^) labels ($L_{\mathrm{Lesion}}^{\mathrm{DPath}}$ and $L_{\mathrm{Pixel}}^{\mathrm{DPath}}$) have high concordance with pathologist labels ($L^{\mathrm{Path}}$). Digital radiologists trained with digital pathologist labels perform with comparable or better accuracy than digital radiologists trained with radiologist or pathologist labels. Moreover, digital radiologists trained with pixel‐level digital pathologist labels ($L_{\mathrm{Pixel}}^{\mathrm{DPath}}$) can enable selective identification of aggressive and indolent cancer components in mixed lesions, which is not possible by radiologists. Evaluation in both cohorts indicate that the digital radiologists trained with digital pathologist labels have generalizable performance in biopsy as well as radical prostatectomy patients. The trend of digital pathologist label‐trained digital radiologists performing better or comparable to human label‐trained digital radiologists is irrespective of the model architecture (Table 3). Thus, digital pathologist labels provide a consistent, standardized, accurate, labor and time‐efficient method for training reliable digital radiologists for selective identification of aggressive and indolent prostate cancer.

Digital pathologist labels not only train the most accurate digital radiologists, but also help overcome the challenges associated with generating human‐annotated pixel‐level histologic grade labels. It is impractical for genitourinary pathologists to manually annotate all prostate pixels with Gleason patterns for a sufficiently large population of patients to train machine learning models. Automated Gleason grading on histopathology images by digital pathologists (a) have excellent performance,^25^, ^36^, ^48^ and (b) have shown to significantly improve Gleason grading by human pathologists.^35^ Digital pathologist labels also improve uniformity in grading by reducing inter‐ and intra‐pathologist variation in Gleason grade group assignment.

Prior studies^16^, ^17^, ^18^, ^19^, ^21^, ^22^, ^23^, ^24^, ^26^, ^41^ ^47^, ^49^ on developing machine learning methods for prostate cancer detection have used different kinds of labels to develop their models. This is the first study to systematically compare and analyze the effect of different labeling strategies on the performance of automated algorithms for prostate cancer detection on MRI (digital radiologists). We trained four different model architectures (U‐Net, branched U‐Net, SPCNet, and the DeepLabv3+) used in prior studies and tested in two independent cohorts to further emphasize that the effect of the labeling strategies is independent of the model type and the dataset used for testing. Our study showed that the SPCNet architecture outperformed the other architectures, irrespective of the label type used for training.

Our study has five noteworthy limitations. First, unlike prior studies,^24^ the number of patients in cohort C1 is relatively small (*N* = 115), primarily due to its uniqueness including registered MRI and histopathology images of radical prostatectomy patients, pixel‐level radiologist and pathologist labels, as well as pixel‐level digital pathologist labels. Despite its small size, the generalizable performance of the deep learning models on the independent cohort C2 indicate the utility of the dataset. Second, all patients in this study are from a single institution (Stanford University) and single manufacturer (GE Healthcare). Third, our study includes retrospective data and has not been used in prospective evaluation. Fourth, the digital pathologist was trained on prostate biopsy histopathology samples,^25^ but was used to generate pixel‐level histologic grade labels on whole‐mount histopathology images. Despite being trained on biopsy histopathology images, the digital pathologist showed high agreement with the human pathologist on the whole‐mount images. Finally, registration errors (~2 mm on the prostate border and 3 mm inside the prostate) in the MRI‐histopathology registration platform^31^ may affect small lesions. Excluding lesions of volumes 250 mm^3^ (6 mm × 6 mm × 6 mm) helps focus on aggressive cancer, as small lesions are not deemed to be clinically significant^45^, ^46^ while helping counter the MRI‐histopathology registration errors in cohort C1. Automated MRI‐histopathology registration is a challenging task due to several factors including the difference in acquisition procedures of radiology and histopathology images, differences in image resolution, slice thickness, and possible changes that may occur between pre‐operative MRI and radical prostatectomy. Despite these challenges, our automated MRI‐histopathology approach provides an accurate labor and time‐efficient approach to map pathology labels onto MRI, enabling the training of digital radiologists with the most accurate ground truth labels.

Identifying and treating aggressive cancer, and reducing over‐treatment of indolent cancer are the primary goals of prostate cancer care. A digital radiologist can help standardize radiologist interpretations, and assist clinicians in reliably detecting and localizing aggressive and indolent cancer on prostate MRI. In order to develop a reliable digital radiologist, it is imperative to train it with the best possible labels. Our experiments show that digital pathologist labels are the best way to train digital radiologists not only because they help develop the most accurate digital radiologist models, but also because they circumvent the challenges associated with acquiring pixel‐level human‐annotated histologic grade labels. A reliable digital radiologist can help prostate cancer care by (1) standardizing radiologist interpretations, (2) helping detect and target aggressive cancers that are currently missed, (3) helping reduce unnecessary invasive biopsies in men without cancer or with indolent cancer, and (4) helping reduce the number of biopsies to detect aggressive cancers by localizing the aggressive cancer components in mixed lesions.

## 5 CONCLUSION

Digital pathologist labels generated by deep learning algorithms on prostate histopathology images can help bridge the gap between prostate radiology and pathology by enabling the training of reliable machine learning models, referred to here as digital radiologists, for selective identification of aggressive and indolent prostate cancer on MRI. Digital pathologists have similar performance to pathologists in selective identification of aggressive and indolent prostate cancer on prostate histopathology images. Digital pathologist‐trained digital radiologists (1) enable selective identification of aggressive and indolent cancer on prostate MRI on a lesion level as well as on a pixel level (which is not possible with any human‐annotated label type), (2) perform better than radiologist‐trained models, (3) perform equally well or better than pathologist label‐trained models, and (3) circumvent the labor, time, and variability challenges associated with human annotations for training digital radiologist models.

## CONFLICT OF INTEREST

Mirabela Rusu has research grants from GE Healthcare and Philips Healthcare.

## Supporting information

Supporting Information

## Data Availability

The data that support the findings of this study are available from the corresponding authors upon reasonable request.
