# Supplementary material for: Bridging the gap between prostate radiology and pathology through machine learning
Source: Med Phys. 2022 Jun 13;49(8):5160–81. doi: 10.1002/mp.15777 (PMC9543295; doi:10.1002/mp.15777)
Supplement: Supplementary file 1 — Supporting Information [file MP-49-5160-s001.pdf]

Bridging the gap between prostate radiology and pathology through machine learning

Supplementary Material

I. MRI parameter acquisition characteristics

Table S1 shows the MRI parameter acquisition characteristics.

II. Data preprocessing

II.A. Registration

For cohort C1, pre-operative MRI and post-operative histopathology images were registered using the previously validated and published **RA**diology **P**athology **S**patial **O**pen-Source multi-**D**imensional **I**ntegration (RAPSODI) registration platform[1]. The RAPSODI registration platform first reconstructs the three-dimensional (3D) histopathology volume, and then registers corresponding histopathology and MRI slices. In RAPSODI, the MRI-histopathology slice-to-slice alignment is achieved by optimizing affine and deformable transforms using a registration based on a three-layered multi-resolution pyramid. RAPSODI achieves a Dice coefficient of 0.97±0.01 for the prostate, a Hausdorff distance of 1.99±0.70 mm for the prostate boundary, a urethra deviation of 3.09±1.45 mm, and a landmark deviation of 2.80±0.59 mm between registered histopathology images and MRI. More details about the registration process are available in the article by Rusu et al.[1]. This MRI-histopathology registration allows accurately mapping the extent of cancer from histopathology images onto MRI.

In addition to MRI-histopathology registration, for cohort C1, T2w and ADC images were manually registered using affine transformations.

II.B. Resampling

The T2w and ADC images of all subjects from both cohorts were cropped around the prostate and resampled to have the same pixel-size (0.29mm x 0.29mm) and the same X-Y dimensions (224x224), similar to our prior studies[2,3,4].

II.C. MRI Intensity Standardization and Intensity Normalization

T2w and ADC image-intensities were standardized using a histogram alignment approach[5] using average histograms derived from the training set of each MRI sequence independently. Standardized MRI intensities were then z-score normalized, similar to our prior studies[2,3].

III. Model Architectures

SPCNet: SPCNet[2] is an architecture based on the hierarchical Holistically-Nested Edge Detector (HED) model[6] that was designed to leverage multiple scales of input features for edge detection. SPCNet has 2 separate encoders for T2w and ADC images respectively, with each encoder taking in three adjacent MRI slices. The outputs from each encoder are concatenated and go through more convolutional layers. Then, the outputs of those convolutional layers are fused with side outputs from both encoders as well as from the post-concatenation convolutional layers. This fused final output is used as input to the final softmax layer that predicts the probability of each class for each pixel.

U-Net: U-Net[7] is a commonly used deep learning model for biomedical image segmentation tasks including prostate cancer detection[8,9]. The network architecture of U-Net consists of a traditional "contracting" path of convolution layers, or encoder, followed by an"expanding" mirror set of convolutional layers, known as the decoder, that outputs the segmentation map. In addition to the main path, "skip-connections" between corresponding encoder and decoder layers allow the decoder to utilize additional features of the input directly from the encoder. Three adjacent slices of T2w images and three slices of the corresponding ADC images were input into the U-Net model as image channels with 6 input channels in total.

Branched U-Net (BrU-Net): A variant of the vanilla U-Net architecture, which we call the branched U-Net (BrU-Net), was used in our experiments. The BrU-Net incorporates the changes that SPCNet incorporates to the baseline HED architecture, i.e., BrU-Net has two separate encoders for the T2w and ADC images, with each encoder taking in three adjacent MRI slices. Decoder has identical layers to that of the original U-Net but has skip-connection inputs from both branches.

DeepLabv3+: DeepLabv3+[10] is a deep learning model for semantic segmentation that builds on prior DeepLab architectures by including atruous convolutions, spatial pyramid pooling, and integrating a decoder that is better at segmentating boundary details. The DeepLabv3+ architecture formed the backbone of the FocalNet model for prostate cancer detection and Gleason grade prediction[11]. For our experiments, the encoder of DeepLabv3+ takes as input one slice of T2w and one slice of ADC per example.

IV. Evaluation Methods

Lesion-level evaluation was performed using a sextant-based approach as detailed in our prior studies[2,4], summarized below:

1. True positives and false negatives were assessed using the 90th percentile of the predicted labels within the ground truth lesion. If the 90th percentile of the predicted labels within the ground truth lesion was cancer, the prediction was considered to be a true positive, alternatively it was considered to be a false negative.

2. True negatives and false positives were assessed by splitting the prostate into sextants (Figure S1). A ground truth negative sextant was defined as one with less than 5% ground truth cancer pixels. Predictions were considered to be true negative if the 90th percentile of the predicted labels for a ground truth normal sextant was normal, alternatively the predictions were considered to be false positive. This sextant-based evaluation method takes into account how the most prostate biopsy protocol is performed clinically, with 2 cores from each sextant resulting in 12-core needle samples.

V. Evaluation Metrics

The following metrics were used for analysis:

$$Dice = \frac{2 * TP}{2 * TP + FP + FN}$$

$$Sensitivity = \frac{TP}{TP + FP}$$

$$Specificity = \frac{TN}{TN + FP}$$

where TP are the true positive and FP are the false positive predictions. The Dice coefficient was computed on a pixel-level, wheras the sensitivities and specificites were computed on a lesion-level using the predicted and ground truth labels. In addition, predicted probabilities were used to compute the lesion-level area under the receiver operating characteristics (ROC-AUC) curves.

VI. Results

VI.A. Studying the effect of different labeling strategies on digital radiologist performance

VI.A.1. Quantitative evaluation of UNet, branched UNet and DeepLabv3+

Cohort C1-test: Figures S2, S3 and S4 presents the 4×4 evaluation matrices of U-Net, branched U-Net and DeepLabv3+ digital radiologist models, when evaluated on cohort C1-test.

Cohort C2: Tables S2, S3 and S4 presents the evaluation tables of U-Net, branched U-Net and DeepLabv3+ digital radiologist models, when evaluated on cohort C2 with radiologist labels.

VI.B. Validation set performance of SPCNet-based digital radiologist

The performance of the SPCNet-based digital radiologist model on the validation sets of 5-fold cross validation are presented in Table S5. The validation set of each fold includes 15 different patients. Evaluation was performed with $\mathcal{L}^{Path}$ labels. We note that for most folds, models trained with digital pathologist models achieve higher ROC-AUCs thatn models trained with radiologist labels.

1 M. Rusu et al., Registration of presurgical MRI and histopathology images from radical prostatectomy via RAPSODI, Medical Physics **47(9)**, 4177 – 4188 (2020).

2 A. Seetharaman et al., Automated detection of aggressive and indolent prostate cancer on magnetic resonance imaging, Medical Physics. (2021).

3 I. Bhattacharya et al., CorrSigNet: Learning correlated prostate cancer signatures from radiology and pathology images for improved computer aided diagnosis, in International Conference on Medical Image Computing and Computer-Assisted Intervention, pages 315–325, Springer, 2020.

4 I. Bhattacharya et al., Selective identification and localization of indolent and aggressive prostate cancers via CorrSigNIA: an MRI-pathology correlation and deep learning framework, Medical image analysis , 102288 (2021).

5 L. G. Nyúl, J. K. Udupa, and X. Zhang, New variants of a method of MRI scale standardization, IEEE Transactions on Medical Imaging **19**, 143–150 (2000).

6 S. Xie and Z. Tu, Holistically-nested edge detection, in Proceedings of the IEEE international conference on computer vision, pages 1395–1403, 2015.

7 O. Ronneberger, P. Fischer, and T. Brox, U-net: Convolutional networks for biomedical image segmentation, in International Conference on Medical image computing and computer-assisted intervention, pages 234–241, Springer, 2015.

8 P. Schelb et al., Classification of cancer at prostate MRI: deep learning versus clinical PI-RADS assessment, Radiology **293**, 607–617 (2019).

9 J. Sanyal, I. Banerjee, L. Hahn, and D. Rubin, An Automated Two-step Pipeline for Aggressive Prostate Lesion Detection from Multi-parametric MR Sequence, AMIA Summits on Translational Science Proceedings **2020**, 552 (2020).

10 L.-C. Chen, Y. Zhu, G. Papandreou, F. Schroff, and H. Adam, Encoder-decoder with atrous separable convolution for semantic image segmentation, in Proceedings of the European conference on computer vision (ECCV), pages 801–818, 2018.

[11] R. Cao, A. M. Bajgiran, S. A. Mirak, S. Shakeri, X. Zhong, D. Enzmann, S. Raman, and K. Sung, Joint prostate cancer detection and gleason score prediction in mp-MRI via FocalNet, IEEE Transactions on Medical Imaging **38**, 2496–2506 (2019).

VII. List of supplementary figure legends

S1. Sextants for lesion-level evaluation. (a) Axial, (b) Sagittal, and (c) Coronal views. The sextant-based approach for evaluating lesions was based on how biopsies are performed clinically. It is very common to do 12-core needle sampling with 2 cores from each sextant.

S2. Quantitative comparison between **UNet digital radiologist** predictions when trained and evaluated using different label types in cohort C1-test. The top row shows results for cancer detection, while the bottom row shows results for aggressive cancer detection. Darker blue boxes in the 4×4 matrices represent higher evaluation metrics.

S3. Quantitative comparison between **branched UNet digital radiologist** predictions when trained and evaluated using different label types in cohort C1-test. The top row shows results for cancer detection, while the bottom row shows results for aggressive cancer detection. Darker blue boxes in the 4×4 matrices represent higher evaluation metrics.

S4. Quantitative comparison between **DeeLabv3+ digital radiologist** predictions when trained and evaluated using different label types in cohort C1-test. The top row shows results for cancer detection, while the bottom row shows results for aggressive cancer detection. Darker blue boxes in the 4×4 matrices represent higher evaluation metrics.

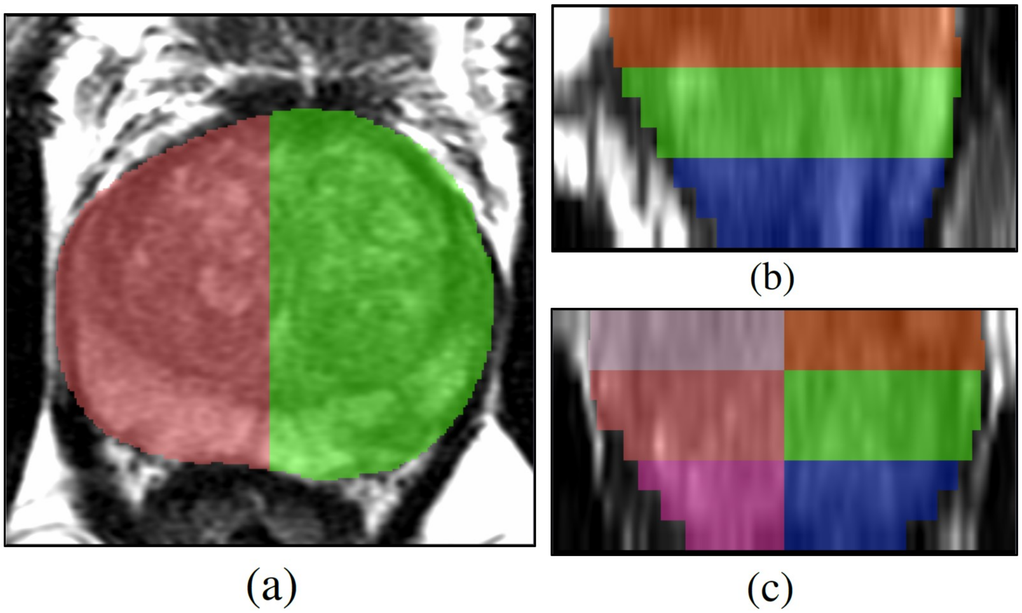

Figure S1: Sextants for lesion-level evaluation. (a) Axial, (b) Sagittal, and (c) Coronal views. The sextant-based approach for evaluating lesions was based on how biopsies are performed clinically. It is very common to do 12-core needle sampling with 2 cores from each sextant.

1

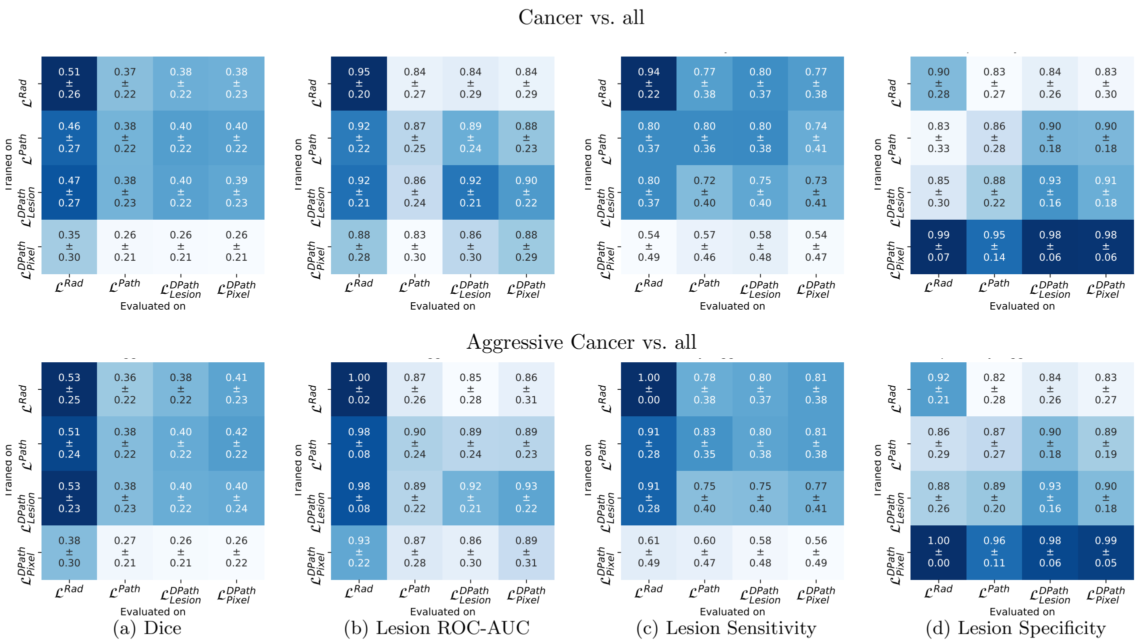

Figure S2: Quantitative comparison between **UNet digital radiologist** predictions when trained and evaluated using different label types in cohort C1-test. The top row shows results for cancer detection, while the bottom row shows results for aggressive cancer detection. Darker blue boxes in the 4×4 matrices represent higher evaluation metrics.

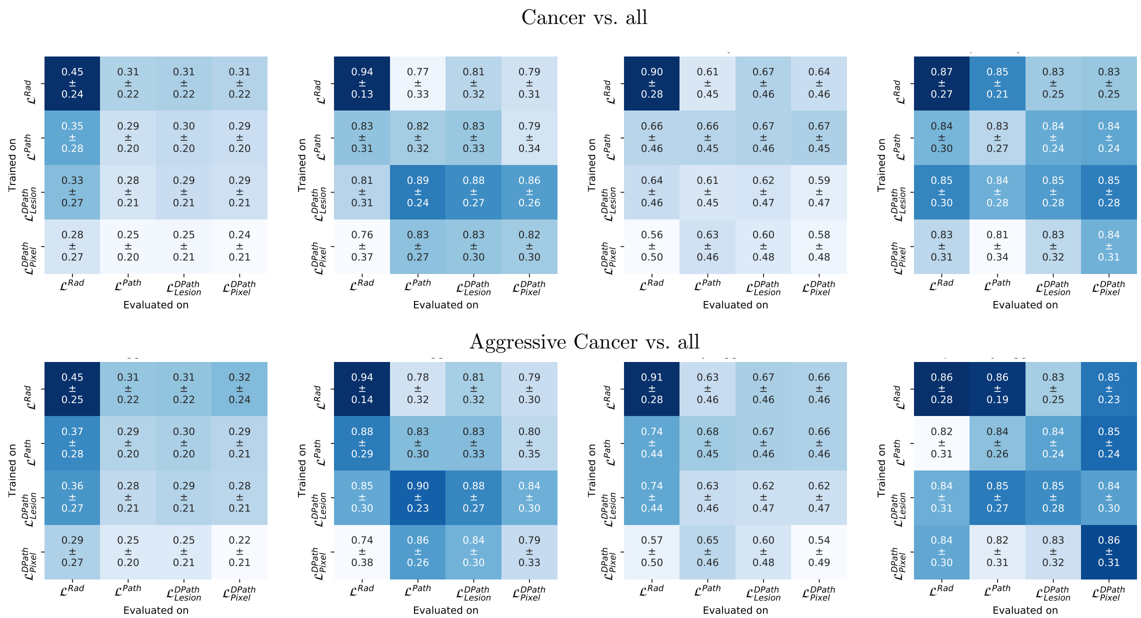

(a) Dice (b) Lesion ROC-AUC (c) Lesion Sensitivity (d) Lesion Specificity

Figure S3: Quantitative comparison between **branched UNet digital radiologist** predictions when trained and evaluated using different label types in cohort C1-test. The top row shows results for cancer detection, while the bottom row shows results for aggressive cancer detection. Darker blue boxes in the 4×4 matrices represent higher evaluation metrics.

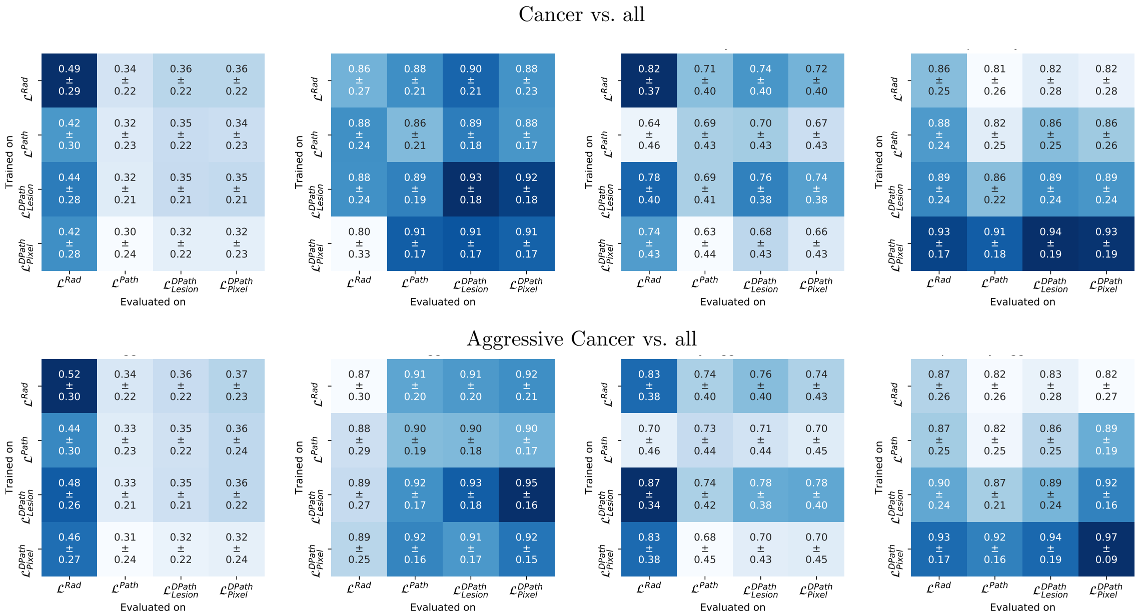

(a) Dice (b) Lesion ROC-AUC (c) Lesion Sensitivity (d) Lesion Specificity

Figure S4: Quantitative comparison between **DeeLabv3+ digital radiologist** predictions when trained and evaluated using different label types in cohort C1-test. The top row shows results for cancer detection, while the bottom row shows results for aggressive cancer detection. Darker blue boxes in the 4×4 matrices represent higher evaluation metrics.

VIII. Supplementary tables

Table S1: Description of MRI parameter acquisition characteristics in our two cohorts.

MRI Statistics	Cohort C1	Cohort C2
T2w		
Repetition Time (TR) (s)	3.9-6.3	2.0-7.4
Echo Time (TE) (ms)	122-130	92-150
Pixel Size (mm)	0.27-0.94	0.39-0.47
Distance between Slices (mm)	3.00-4.20	3.00-4.20
No. of Slices	24-43	20-43
ADC		
b-values (s/mm^2)	[0, 50, 800, 1000, 1200]	[0, 25, 50, 800, 1200, 1400]
Pixel Size (mm)	0.78-1.50	0.78-1.01
Distance between Slices (mm)	3.00-5.20	3.00-4.60
No. of Slices	15-40	14-42

Table S2: Lesion-level evaluation in cohort C2 of the different **UNet** models trained using cohort C1-train. Cohort C2 only had biopsy-confirmed radiologist labels ($\mathcal{L}^{Rad}$), thus all evaluations were with respect to $\mathcal{L}^{Rad}$.

Trained with Label Type	AUC-ROC	Dice	Sens.	Spec.
**Cancer vs. all (N = 160, number of lesions = 193)**				
$\mathcal{L}^{Rad}$	**0.82±0.31**	**0.39±0.26**	0.76±0.39	0.67±0.38
$\mathcal{L}^{Path}$	0.80±0.32	0.37±0.25	**0.80±0.35**	0.63±0.40
$\mathcal{L}^{DPath}_{Lesion}$	0.78±0.35	0.36±0.25	0.78±0.38	0.65±0.38
$\mathcal{L}^{DPath}_{Pixel}$	0.82±0.32	0.31±0.26	0.62±0.45	**0.85±0.28**
**Aggressive Cancer vs. all (N = 160, number of lesions = 132)**				
$\mathcal{L}^{Rad}$	0.78±0.25	**0.25±0.19**	**0.89±0.31**	0.47±0.34
$\mathcal{L}^{Path}$	0.67±0.39	0.25±0.24	0.63±0.48	0.44±0.32
$\mathcal{L}^{DPath}_{Lesion}$	0.70±0.39	0.21±0.20	0.55±0.48	0.51±0.34
$\mathcal{L}^{DPath}_{Pixel}$	**0.79±0.34**	0.23±0.21	0.66±0.46	**0.70±0.28**
**Indolent Cancer vs. all (N = 160, number of lesions = 61)**				
$\mathcal{L}^{Rad}$	0.50±0.44	0.00±0.01	0.00±0.00	**1.00±0.00**
$\mathcal{L}^{Path}$	0.39±0.41	0.00±0.00	0.00±0.00	**1.00±0.00**
$\mathcal{L}^{DPath}_{Lesion}$	0.40±0.40	0.00±0.00	0.00±0.00	**1.00±0.00**
$\mathcal{L}^{DPath}_{Pixel}$	**0.72±0.33**	**0.02±0.04**	**0.04±0.18**	**1.00±0.00**

Table S3: Lesion-level evaluation in cohort C2 of the different **branched UNet** models trained using cohort C1-train. Cohort C2 only had biopsy-confirmed radiologist labels ($\mathcal{L}^{Rad}$), thus all evaluations were with respect to $\mathcal{L}^{Rad}$.

Cancer vs. all (N = 160, number of lesions = 193)				
Trained with Label Type	AUC-ROC	Dice	Sens.	Spec.
$\mathcal{L}^{Rad}$	**0.82±0.33**	**0.38±0.26**	**0.80±0.38**	**0.68±0.35**
$\mathcal{L}^{Path}$	0.78±0.35	0.36±0.25	0.77±0.40	0.65±0.39
$\mathcal{L}^{DPath}_{Lesion}$	0.77±0.35	0.36±0.25	0.77±0.40	0.65±0.39
$\mathcal{L}^{DPath}_{Pixel}$	0.75±0.36	0.33±0.25	0.70±0.43	0.67±0.38
Aggressive Cancer vs. all (N = 160, number of lesions = 132)				
Trained with Label Type	AUC-ROC	Dice	Sens.	Spec.
$\mathcal{L}^{Rad}$	**0.86±0.30**	**0.42±0.25**	**0.86±0.33**	0.66±0.36
$\mathcal{L}^{Path}$	0.85±0.30	0.40±0.24	0.84±0.35	0.66±0.40
$\mathcal{L}^{DPath}_{Lesion}$	0.83±0.32	0.39±0.24	0.81±0.38	0.61±0.38
$\mathcal{L}^{DPath}_{Pixel}$	0.80±0.33	0.36±0.24	0.75±0.42	**0.68±0.37**
Indolent Cancer vs. all (N = 160, number of lesions = 61)				
Trained with Label Type	AUC-ROC	Dice	Sens.	Spec.
$\mathcal{L}^{Rad}$	0.51±0.44	0.00±0.00	0.00±0.00	1.00±0.00
$\mathcal{L}^{Path}$	0.56±0.42	0.00±0.00	0.00±0.00	**1.00±0.00**
$\mathcal{L}^{DPath}_{Lesion}$	**0.64±0.38**	0.00±0.00	0.00±0.00	**1.00±0.00**
$\mathcal{L}^{DPath}_{Pixel}$	0.59±0.44	**0.10±0.15**	**0.24±0.41**	0.94±0.18

Table S4: Lesion-level evaluation in cohort C2 of the different **DeepLabv3+** models trained using cohort C1-train. Cohort C2 only had biopsy-confirmed radiologist labels ($\mathcal{L}^{Rad}$), thus all evaluations were with respect to $\mathcal{L}^{Rad}$.

Cancer vs. all (N = 160, number of lesions = 193)				
Trained with Label Type	AUC-ROC	Dice	Sens.	Spec.
$\mathcal{L}^{Rad}$	0.81±0.34	**0.39±0.27**	**0.75±0.41**	0.75±0.35
$\mathcal{L}^{Path}$	**0.81±0.32**	0.35±0.25	0.73±0.41	0.71±0.37
$\mathcal{L}^{DPath}_{Lesion}$	0.79±0.33	0.34±0.25	0.69±0.42	0.73±0.36
$\mathcal{L}^{DPath}_{Pixel}$	0.80±0.33	0.31±0.26	0.61±0.45	**0.86±0.29**
Aggressive Cancer vs. all (N = 160, number of lesions = 132)				
Trained with Label Type	AUC-ROC	Dice	Sens.	Spec.
$\mathcal{L}^{Rad}$	**0.86±0.30**	**0.44±0.24**	**0.83±0.35**	0.72±0.37
$\mathcal{L}^{Path}$	0.86±0.27	0.39±0.24	0.78±0.39	0.71±0.38
$\mathcal{L}^{DPath}_{Lesion}$	0.86±0.28	0.39±0.25	0.77±0.39	0.73±0.37
$\mathcal{L}^{DPath}_{Pixel}$	0.85±0.31	0.37±0.26	0.69±0.43	**0.84±0.31**
Indolent Cancer vs. all (N = 160, number of lesions = 61)				
Trained with Label Type	AUC-ROC	Dice	Sens.	Spec.
$\mathcal{L}^{Rad}$	**0.70±0.38**	0.00±0.00	0.01±0.07	**1.00±0.00**
$\mathcal{L}^{Path}$	0.55±0.40	0.00±0.00	0.00±0.00	**1.00±0.00**
$\mathcal{L}^{DPath}_{Lesion}$	0.58±0.37	0.00±0.00	0.00±0.00	**1.00±0.00**
$\mathcal{L}^{DPath}_{Pixel}$	0.61±0.42	**0.07±0.14**	**0.22±0.40**	0.96±0.12

Table S5: Validation set performances of the SPCNet-based digital radiologist models in 5-fold cross validation. Each individual fold model was validated with 15 different patients using the sextant-based evaluation method. The validation set performance shows the same trends as in the independent test sets, with digital pathologist label-trained models achieving higher or similar ROC-AUCs and Dice coefficient when compared to other label-trained models.

**Cancer vs. all (Number of patients in each fold = 15)**										
	Fold 0, lesions = 17, neg sextants = 38		Fold 1, lesions = 17, neg sextants = 45		Fold 2, lesions = 10, neg sextants = 40		Fold 3, lesions = 17, neg sextants = 33		Fold 4, lesions = 17, neg sextants = 55	
Trained with Label type	AUC-ROC	Dice	AUC-ROC	Dice	AUC-ROC	Dice	AUC-ROC	Dice	AUC-ROC	Dice
$\mathcal{L}^{Rad}$	0.78±0.33	**0.40±0.23**	0.78±0.37	0.27±0.22	0.84±0.18	0.16±0.06	0.92±0.16	0.38±0.21	0.78±0.26	0.26±0.24
$\mathcal{L}^{Path}$	0.76±0.33	0.32±0.20	0.74±0.42	0.31±0.20	**0.95±0.10**	0.24±0.11	**0.96±0.13**	0.37±0.15	0.80 ±0.26	0.26±0.26
$\mathcal{L}^{DPath}_{Lesion}$	**0.81±0.29**	0.34±0.20	0.80±0.36	**0.34±0.21**	**0.95±0.10**	**0.20±0.11**	0.92±0.18	0.33±0.12	0.80±0.26	**0.31±0.24**
$\mathcal{L}^{DPath}_{Pixel}$	0.68±0.36	0.250.22	**0.81±0.31**	0.270.24	0.86±0.18	0.180.07	0.92±0.18	**0.39±0.15**	**0.83±0.30**	0.29±0.24
**Agg cancer vs. all (Number of patients in each fold = 15)**										
	Fold 0, lesions = 16, neg sextants = 39		Fold 1, lesions = 16, neg sextants = 43		Fold 2, lesions = 10, neg sextants = 40		Fold 3, lesions = 17, neg sextants = 33		Fold 4, lesions = 17, neg sextants = 55	
Trained with Label type	AUC-ROC	Dice	AUC-ROC	Dice	AUC-ROC	Dice	AUC-ROC	Dice	AUC-ROC	Dice
$\mathcal{L}^{Rad}$	0.82±0.32	**0.38±0.22**	0.80±0.38	0.29±0.22	0.84±0.18	0.16±0.06	0.92±0.16	0.42±0.19	0.78±0.26	0.26±0.24
$\mathcal{L}^{Path}$	0.80±0.33	0.31±0.19	0.80±0.38	0.33±0.22	**0.95±0.10**	**0.24±0.11**	**0.95±0.14**	0.38±0.16	0.80±0.26	0.26±0.26
$\mathcal{L}^{DPath}_{Lesion}$	**0.83±0.30**	0.33±0.22	0.82±0.36	**0.37±0.20**	**0.95±0.10**	0.20±0.11	0.89±0.23	0.36±0.11	0.80±0.26	**0.31±0.24**
$\mathcal{L}^{DPath}_{Pixel}$	0.68±0.37	0.25±0.22	**0.83±0.31**	0.29±0.23	0.86±0.18	0.18±0.07	**0.95±0.14**	**0.42±0.11**	**0.83±0.30**	0.29±0.24
